# A network approach to investigating the inter-relationship between health-related quality of life dimensions and depression in 1735 Chinese patients with heterogeneous cancers

**DOI:** 10.3389/fpubh.2023.1325986

**Published:** 2024-01-23

**Authors:** Sulaiman Muhetaer, Peierdun Mijiti, Kaibinuer Aierken, Huang Ziyin, Wulan Talapuhan, Kaibinuer Tuoheti, Ye Lixia, Qi Shuang, Wei Jingjing

**Affiliations:** ^1^Department of Epidemiology and Biostatistics, School of Public Health, Xinjiang Medical University, Urumqi, Xinjiang, China; ^2^Department of Gynecological Radiotherapy, The Third Hospital Affiliated to Xinjiang Medical University (Affiliated Cancer Hospital), Urumqi, Xinjiang, China; ^3^Department of Health Policy and Management, School of Public Health, Xinjiang Medical University, Urumqi, Xinjiang, China

**Keywords:** health-related quality of life, depression, network analysis, directed acyclic graph, inter-correlation

## Abstract

**Background:**

We aimed to explore the inter-connection between depression and HRQOL dimensions in cancer patients using a network approach, which might provide new insights for precise interventions to improve cancer patients’ overall HRQOL.

**Methods:**

Between June 1, 2016, and August 31, 2017, a total of 1735 eligible patients with heterogeneous types of cancer were recruited. The Zung Self-Rating Depression Scale (SDS) and the European Organization for Research and Treatment of Cancer Quality of Life Questionnaire (EORTC QLQ-C30) were used to measure patients’ depression status and HRQOL, respectively. A regularized partial correlation network was established. Central and bridge symptoms/functions were identified using expected influence and bridge expected influence. A directed acyclic graph (DAG) was used to explore the possible causal relationship between depression and HRQOL dimensions.

**Results:**

In this study, depression and 15 dimensions of the EORTC-QLQ-C30 scale were highly inter-correlated and could be represented as a network. We found that nearly two-thirds of cancer patients experienced various degrees of depression, and depression was consistently the central symptom in the network, in addition to nausea/vomiting, pain, and physical function. DAG and bridge symptoms indicated that depression might influence overall HRQOL in cancer patients mainly through emotional function, pain, physical function, and sleeplessness, particularly in cancer patients with moderate-to-severe depression. The disparity in network structures between mild and moderate-to-severe depression suggested that the relationship between depression and HRQOL dimensions might be bidirectional.

**Conclusion:**

The prevalence of depression remained high in Chinese patients with cancer, and depression may influence various symptoms and functions within the HRQOL network. Screening and early treatment of depression were warranted to improve the overall HRQOL of cancer patients, in addition to adequate treatment of pain and nausea/vomiting and improvement in physical function.

## 1 Introduction

Cancer remains one of the most significant public health issues globally (1). Although the incidence and mortality remain high in most countries, improvements in treatment, cancer prevention, and early detection have led to a growing prevalence of cancer survivors (2). As more and more cancer patients live longer, the health-related quality of life (HRQOL) of cancer survivors has become equally important (3). Multiple factors, including demographic characteristics of cancer patients, cancer type, treatment and its side effects, comorbidity, emotional disorders, social support, etc. may impact the HRQOL of cancer survivors (3–5). Among them, emotional disorders were often overlooked although their prevalence among cancer patients was high (6). Depression was one of the most common emotional disorders among cancer patients (7) with a prevalence of 7.9–32.4% (8). There was a wealth of evidence indicating a strong correlation between depression and overall HRQOL of cancer patients (9, 10). However, HRQOL is a multidimensional construct encompassing multiple symptoms and functions, and these functions/symptoms might correlate and interact with each other. The exact nature of the complex inter-correlations between depression and HRQOL symptoms/functions and their causal mechanisms is still unknown.

Network analysis offers a new perspective in this regard. Network analysis is a method to study the relationships, interactions, and structure of various elements within a system (11). By constructing a symptom network and identifying symptoms with high centrality and their correlation, we may find the most influential symptoms in the network and manage symptoms more precisely (12, 13). In addition, the network analysis can be used to assess the complex interactions between symptoms of comorbid psychiatric disorders (e.g., depression and anxiety) or systems (e.g., quality of life and pain) and identifies the bridge symptoms between them, which are priority targets for clinical intervention (14–16). Network analysis was also applied in psychometric analysis of HRQOL data in previous studies (17–19).

Most network analysis studies used cross-sectional study design and built undirected partial correlation networks, which did not provide information about causal mechanisms. However, recent studies used Bayesian learning and directed acyclic graph (DAG) to explore the potential causal relationships between symptoms (20–23). DAGs can uncover possible directions of conditional dependency relationships between variables and provide important insights into possible causal relationships in cross-sectional designs.

In this study, we aim to investigate the complex interaction between 15 HRQOL dimensions and depression by constructing an HRQOL-depression network in heterogeneous cancer patients, identify the central symptoms/functions in the network and the bridge symptoms/functions connecting HRQOL dimensions and depression, and explore the possible causal relationship between HRQOL dimensions and depression. This may provide implications for precise intervention on the HRQOL of cancer patients with emotional disorders.

## 2 Materials and methods

### 2.1 Study settings and participants

All cancer patients who were admitted to the Third Hospital Affiliated to Xinjiang Medical University (Affiliated Cancer Hospital) between June 1, 2016, and August 31, 2017, and who met the inclusion and exclusion criteria were selected as study participants. The inclusion criteria were cancer patients based on clinical diagnosis (breast, colorectal, cervical, gastric, head and neck, esophagus, and lung cancers), aged 18 years or older, and providing written consent. The exclusion criteria were being unable to complete the self-rating scale, incomplete medical records, no evaluation of HRQOL and depression, and written consent not being obtained.

### 2.2 Data collection

We extracted basic and clinical data from patients’ medical records; these data included age, gender, cancer types, time since first diagnosis, tumor-node-metastasis (TNM) staging, treatment received since diagnosis (surgery, chemotherapy, radiotherapy), and comorbidity (type-2 diabetes mellitus, chronic heart disease, and hypertension). In addition, the HRQOL and depression status of patients were assessed using the Zung Self-Rating Depression Scale (SDS) and the European Organization for Research and Treatment of Cancer Quality of Life Questionnaire (EORTC QLQ-C30).

### 2.3 Measures

#### 2.3.1 The Zung self-rating depression scale

SDS was developed by William W.K. Zung in 1965 and used to assess an individual’s depressive symptoms in terms of emotions, cognition, and physiology through self-reporting. The scale consists of 20 items, with scores ranging from 1 (none or a little of the time) to 4 (most or all of the time). The final index score was converted by multiplying the raw score by 1.25 and then rounding off decimal places. The severity of depression was categorized according to the index score: nil depression (index score < 50), mild depression (index score 50–59), moderate depression (index score 60–69), and severe depression (index score ≥ 70) (24).

#### 2.3.2 The European organization for research and treatment of Cancer quality of life questionnaire

EORTC QLQ-C30 was used to measure the HRQOL of cancer patients. The questionnaire consists of 15 domains, including five functional domains, namely, physical functioning (PF), role functioning (RF), cognitive functioning (*CF*), emotional functioning (EF), and social functioning (SF); nine symptom domains including dyspnea (DY), nausea and vomiting (NV), loss of appetite (AP), fatigue (FA), pain (PA), sleeplessness (SL), constipation (CO), diarrhea (DI), and financial difficulties (FI); and one global health status (QL). A high functional score represents high HRQOL, and a high symptom score indicates severe physical symptoms (25).

### 2.4 Statistical analysis

#### 2.4.1 Network estimation

The network estimation was completed in R 4.2.1 and the R packages qgraph (26) and mgm (27). We constructed the SDS and HRQOL network which consisted of nodes represented by depression and 15 subscales of EORTC QLQ-C30 using the Gaussian graphical model (GGM) with regularized partial correlations (28). The nodes in the network are interconnected by edges, which signify a regularized partial correlation between two nodes while controlling for all other nodes in the network. Each edge is assigned a weight that is regularized using a graphical lasso (least absolute shrinkage and selection operator, LASSO) to guarantee the high specificity of the connections. This process leads to the creation of a concise and easily interpretable model (29). Thicker edges represent stronger connections, with blue edges indicating positive connection and red edges indicating negative connection. The layout of the presented networks was based on the Fruchterman-Reingold algorithm (30).

To identify the central (influential) symptoms in the network, the expected influence (EI) was calculated. EI is a more accurate centrality measure that represents the cumulative weight of all its edges, accounting for both positive and negative associations with its neighboring nodes in the network. In other words, EI accounts for the sign of the association between two nodes (i.e., negative or positive partial correlation) by summing the absolute values of the edges connected to the node (31). Additionally, the role of symptoms/functions as bridges between depression and HRQOL dimensions was assessed using the bridge expected influence (bEI) of each symptom/dimension. The bEI of a node was determined by summing the edge weights to the nodes of all other symptoms, indicating the importance of an individual symptom in linking different clusters of disorders or systems (32).

#### 2.4.2 Network stability

To assess the accuracy of edge weights, the 95% confidence interval was plotted for each edge in the presented networks, using 1,000 bootstrap samples. The stability of EI and bEI was evaluated by calculating the Correlation Stability (CS) coefficient through a case-dropping bootstrap approach (1,000 times). As per the recommended guidelines (27), an ideal CS coefficient is above 0.5 and should not fall below 0.25. Furthermore, bootstrapped difference tests were conducted for both edge weights, EI and bEI (1,000 times). All these procedures were performed using the R-package bootnet (28).

#### 2.4.3 Network comparison

We investigated whether the network characteristics differed between gender, age groups, TNM stages, months from diagnosis, depression severity, and treatment types. The Network Comparison Test (NCT) was performed to assess differences in the network structure, global strength, and each edge between the two networks using Holm-Bonferroni correction of *p*-values due to multiple tests (33). These tests were performed with the R-package NetworkComparisonTest (34).

#### 2.4.4 Directed acyclic graph

DAG can encode the conditional independence relationships between nodes in cross-sectional data and identify acceptable causal relationships among them. The R package bnlearn and the Bayesian hill-climbing algorithm were used (35, 36). The algorithm calculates the structure of the network model by adding, deleting, and reversing the direction of edges, ultimately optimizing the goodness-of-fit score (i.e., the Bayesian information criterion). To ensure the stability of the resulting DAG, we then bootstrapped 10,000 samples (with replacement). When determining the direction of each edge, if the direction of the directed edge is present in more than 51% of the DAGs in the 10,000 bootstraps, the directional edges would be represented in the final DAG (37).

Statistical analysis was performed using R 4.2.1. Categorical variables were expressed as proportions or percentages. Mean ± standard deviation (x̅ ± s) was used to describe normally distributed continuous variables. One-way ANOVA was used to compare normally distributed continuous variables between multiple independent groups, and the Bonferroni method was used for pairwise comparisons. Pearson’s correlation was used to examine the relationship between depression severity and HRQOL dimensions. A *value of p* of less than 0.05 was considered statistically significant.

## 3 Results

### 3.1 Characteristics of participants

Among 1864 cancer patients who were admitted to The Third Hospital Affiliated to Xinjiang Medical University (Affiliated Cancer Hospital) during the study period, 1735 met the inclusion and exclusion criteria (112 patients refused to participate in the study, and 17 patients had incomplete medical records) and were included in the final analysis. The mean age of the eligible patients was 53.91 ± 11.42 years, ranging from 18 to 85 years, and 67.55% (1,172/1735) were women. A total of 1,217 (70.14%) were diagnosed with cancer for less than 12 months. The most prevalent cancer type in the 1735 patients was breast cancer (26.6%, 462/1735), followed by colorectal cancer (17.1%, 297/1735), cervical cancer (16.5%, 287/1735), gastric cancer (12.5%, 217/1735), head and neck cancer (11.0%, 191/1735), esophagus cancer (10.9%, 189/1735), and lung cancer (5.3%, 92/1735). The characteristics of cancer patients are shown in Table 1.

**Table 1:** Basic characteristics of cancer patients (*n*,%).

	Total (*n* = 1735)	Breast (*n* = 462)	Colorectal (*n* = 297)	Cervical (*n* = 287)	Gastric (*n* = 217)	Head and neck (*n* = 191)	Esophagus (*n* = 189)	Lung (*n* = 92)
Sex								
Men	563(32.45)	6(1.30)	175(58.92)	3(1.05)	159(73.27)	35(18.32)	124(65.61)	61(66.30)
Woman	1,172(67.55)	456(98.7)	122(41.08)	284(98.95)	58(26.73)	156(81.68)	65(34.39)	31(33.70)
Age								
<65	1,407(81.10)	424(91.77)	218(73.40)	248(86.41)	157(72.35)	171(89.53)	132(69.84)	57(61.96)
≥65	328(18.90)	38(8.23)	79(26.60)	39(13.59)	60(27.65)	20(10.47)	57(30.16)	35(38.04)
Months from diagnosis, median(range)	3.31[0.66,15.10]	7.93[1.81,27.51]	3.87[0.69,17.41]	1.02[0.20,3.44]	2.59[0.43,7.87]	5.41[0.84,23.43]	2.89[0.49,9.41]	3.36[0.80,14.32]
<12 Month	1,217(70.14)	266(57.58)	203(68.35)	241(83.97)	172(79.26)	125(65.45)	146(77.25)	64(69.57)
≥12 Month	518(29.86)	196(42.42)	94(31.65)	46(16.03)	45(20.74)	66(34.55)	43(22.75)	28(30.43)
TNM Staging								
T1-T2	772(44.5)	296(64.07)	104(35.02)	177(61.67)	52(23.96)	73(38.22)	50(26.46)	20(21.74)
T3-T4	963(55.5)	166(35.93)	193(64.98)	110(38.33)	165(76.04)	118(61.78)	139(73.54)	72(78.26)
Surgery								
Yes	1,183(68.18)	451(97.62)	233(78.45)	108(37.63)	130(59.91)	150(78.53)	84(44.44)	27(29.35)
No	552(31.82)	11(2.38)	64(21.55)	179(62.37)	87(40.09)	41(21.47)	105(55.56)	65(70.65)
Chemotherapy								
Yes	1,254(72.28)	415(89.83)	210(70.71)	187(65.16)	172(79.26)	98(51.31)	100(52.91)	72(78.26)
No	481(27.72)	47(10.17)	87(29.29)	100(34.84)	45(20.74)	93(48.69)	89(47.09)	20(21.74)
Radiotherapy								
Yes	655(37.75)	226(48.92)	57(19.19)	215(74.91)	13(5.99)	51(26.70)	64(33.86)	29(31.52)
No	1,080(62.25)	236(51.08)	240(80.81)	72(25.09)	204(94.01)	140(73.3)	125(66.14)	63(68.48)
Any comorbidities*								
Yes	321(18.50)	56(12.12)	68(22.90)	66(23.00)	48(22.12)	30(15.71)	28(14.81)	25(27.17)
No	1,414(81.50)	406(87.88)	229(77.10)	221(77.00)	169(77.88)	161(84.29)	161(85.19)	67(72.83)
Depression								
No	568(32.74)	217(46.97)	126(42.42)	56(19.51)	61(28.11)	67(35.08)	23(12.17)	18(19.57)
Mild	564(32.51)	165(35.71)	95(31.99)	88(30.66)	64(29.49)	60(31.41)	61(32.28)	31(33.70)
Moderate	465(26.80)	68(14.72)	58(19.53)	115(40.07)	62(28.57)	51(26.70)	77(40.74)	34(36.96)
Severe	138(7.95)	12(2.60)	18(6.06)	28(9.76)	30(13.82)	13(6.81)	28(14.81)	9(9.78)

*Type 2 diabetes mellitus, and/or chronic heart disease, and/or hypertension.

### 3.2 Depression and HRQOL subscale scores in patients with seven cancer types

The overall prevalence of depression in cancer patients was 67.3% (1,167/1735). A higher prevalence of depression was observed among patients with cervical (80.5%), esophagus (87.8%), and lung cancers (80.4%). The prevalence of other common symptoms in cancer patients is shown in Supplementary Table S1. The mean scores of SL and FA were higher in all and specific cancer patients compared to other symptoms, indicating they had higher sleeplessness and fatigue burden. The mean scores of EF and SF were lower in all and specific cancer patients compared to other function scores, indicating they had worse social and emotional functions (Table 2). The associations and correlations between HRQOL dimensions and severity of depression are shown in Table 3. All dimensions of HRQOL except DI were associated with the severity of depression. The severity of depression was positively correlated with all symptom dimensions of HRQOL except DI (*r* ranged from 0.241 to 0.509, all *p* < 0.001) and negatively correlated with all functions of HRQOL (*r* ranged from −0.561 to-0.382, all *p* < 0.001).

**Table 2:** Mean scores and SDs of depression and EORTC-QLQ-C30 symptoms/functions scales by cancer type.

Symptoms/Functions	total (*n* = 1735)	Breast (*n* = 462)	Colorectal (*n* = 297)	Cervical (*n* = 287)	Gastric (*n* = 217)	Head and neck* (*n* = 191)	Esophagus (*n* = 189)	Lung (*n* = 92)
Depression	55.66 ± 10.98	51.29 ± 9.90	52.98 ± 10.83	59.72 ± 9.72	57.76 ± 11.49	54.73 ± 11.23	61.20 ± 9.37	59.13 ± 10.18
PF	74.6 ± 17.47	80.09 ± 13.78	76.30 ± 17.03	71.06 ± 16.10	72.23 ± 18.49	77.73 ± 16.89	67.11 ± 20.79	67.05 ± 19.68
RF	79.88 ± 21.34	88.17 ± 18.92	80.25 ± 20.15	75.38 ± 19.02	74.35 ± 22.16	83.17 ± 19.97	72.64 ± 23.10	72.10 ± 25.09
EF	69.83 ± 22.27	75.46 ± 18.88	74.41 ± 22.11	62.40 ± 24.92	68.47 ± 21.16	69.12 ± 22.03	62.79 ± 22.94	69.11 ± 20.77
*CF*	79.51 ± 15.23	82.83 ± 13.35	81.26 ± 15.14	75.32 ± 16.77	77.50 ± 15.1	81.33 ± 15.21	76.46 ± 16.12	77.54 ± 12.94
SF	69.84 ± 19.15	75.05 ± 19.16	71.66 ± 19.62	66.09 ± 16.57	66.05 ± 17.34	72.00 ± 20.75	64.83 ± 18.58	64.13 ± 19.13
QL	63.61 ± 15.81	68.03 ± 15.86	65.94 ± 14.53	61.13 ± 14.93	59.68 ± 15.02	64.2 ± 17.94	57.51 ± 15.11	62.23 ± 13.04
FA	27.69 ± 19.88	21.57 ± 19.26	24.95 ± 18.37	33.1 ± 19.51	32.67 ± 20.95	25.83 ± 19.34	33.10 ± 18.90	31.4 ± 18.75
NV	6.36 ± 13.50	4.08 ± 10.51	5.11 ± 12.07	8.54 ± 14.87	10.29 ± 17.47	4.89 ± 11.70	7.05 ± 13.21	7.43 ± 16.82
PA	15.63 ± 19.42	9.63 ± 13.86	12.29 ± 17.90	22.42 ± 20.46	16.82 ± 21.46	15.27 ± 20.04	23.1 ± 21.97	17.93 ± 21.00
DY	12.81 ± 19.52	11.54 ± 18.67	11.22 ± 18.00	11.27 ± 18.93	13.06 ± 18.94	11.87 ± 18.70	15.17 ± 22.40	25.72 ± 22.16
SL	34.64 ± 29.69	33.69 ± 28.58	33.11 ± 29.25	32.17 ± 30.64	36.25 ± 29.86	33.86 ± 28.51	40.74 ± 31.39	37.32 ± 30.80
AP	15.73 ± 24.52	8.66 ± 18.42	12.91 ± 21.62	20.33 ± 26.76	25.50 ± 29.66	12.91 ± 24.34	21.34 ± 25.90	17.39 ± 24.45
CO	16.96 ± 25.08	12.34 ± 21.27	16.50 ± 24.83	19.05 ± 26.76	22.12 ± 26.3	14.31 ± 23.04	23.10 ± 29.40	15.94 ± 24.94
DI	6.97 ± 16.15	3.39 ± 10.78	10.89 ± 20.44	10.22 ± 19.41	8.76 ± 17.57	5.76 ± 13.95	5.47 ± 13.73	3.62 ± 11.54
FI	29.03 ± 30.05	20.78 ± 28.48	21.89 ± 26.49	44.13 ± 31.96	33.03 ± 29.4	24.43 ± 28.96	39.86 ± 28.53	24.28 ± 23.23

PF: Physical Functioning; RF: Role Functioning; CF: Cognitive Functioning; EF: Emotional Functioning; SF: Social Functioning; FA: Fatigue; NV: Nausea/Vomiting; PA: Pain; DY: Dyspnea; AP: Appetite; SL: Sleeplessness; CO: Constipation; DI: Diarrhea; FI: Financial hardship; QL: General health status.

Head and Neck*: Nasal cancer; Laryngeal cancer; Thyroid cancer; Oral cancer; Tongue cancer; Lip cancer.

**Table 3:** Association of depression severity with HRQOL subscale scores in cancer patients.

Symptoms /Functions	No depression	Mild depression	Moderate depression	Major depression	*F*	*P*	*r*
PF	83.59 ± 10.92^ϯζ^	74.80 ± 13.46*^ζ^	64.25 ± 17.21*^ϯ^	43.59 ± 21.10*^ϯζ^	535.51	<0.001	−0.561**
RF	89.53 ± 16.16^ϯζ^	79.76 ± 18.85*^ζ^	68.74 ± 19.82*^ϯ^	48.41 ± 22.77*^ϯζ^	412.29	<0.001	−0.491**
EF	81.96 ± 16.05^ϯζ^	67.96 ± 19.97*^ζ^	55.02 ± 21.81*^ϯ^	42.11 ± 16.62*^ϯζ^	507.57	<0.001	−0.544**
*CF*	84.92 ± 12.44^ϯζ^	79.42 ± 14.26*^ζ^	72.47 ± 16.26^*ϯ^	64.35 ± 15.70*^ϯζ^	246.10	<0.001	−0.382**
SF	78.45 ± 17.49^ϯζ^	67.84 ± 16.99*^ζ^	60.94 ± 16.66*^ϯ^	49.09 ± 16.97*^ϯζ^	346.89	<0.001	−0.439**
QL	70.80 ± 14.66^ϯζ^	62.03 ± 13.57*^ζ^	56.65 ± 13.86*^ϯ^	44.44 ± 12.16*^ϯζ^	409.42	<0.001	−0.441**
FA	18.03 ± 16.39^ϯζ^	28.02 ± 16.83*^ζ^	39.42 ± 17.64*^ϯ^	56.28 ± 16.17*^ϯζ^	466.15	<0.001	0.509**
NV	2.47 ± 8.62^ϯζ^	5.95 ± 12.52*^ζ^	10.92 ± 15.92*^ϯ^	21.30 ± 21.36*^ϯζ^	238.74	<0.001	0.331**
PA	6.98 ± 12.50^ϯζ^	15.08 ± 17.14*^ζ^	26.42 ± 19.94*^ϯ^	44.91 ± 23.73*^ϯζ^	439.23	<0.001	0.487**
DY	8.67 ± 15.55^ϯζ^	11.90 ± 18.51*^ζ^	17.72 ± 22.43*^ϯ^	31.17 ± 25.49*^ϯζ^	121.77	<0.001	0.241**
SL	25.17 ± 26.54^ϯζ^	39.85 ± 29.01*	40.82 ± 30.81*	51.54 ± 31.04*^ϯζ^	137.69	<0.001	0.289**
AP	6.18 ± 15.94^ϯζ^	14.46 ± 22.20*^ζ^	27.74 ± 27.22*^ϯ^	51.85 ± 27.85*^ϯζ^	391.53	<0.001	0.294**
CO	10.14 ± 19.02^ϯζ^	15.70 ± 24.32*^ζ^	24.89 ± 27.84*^ϯ^	46.60 ± 28.80*^ϯζ^	207.25	<0.001	0.283**
DI	6.27 ± 15.32^ζ^	6.69 ± 15.46	8.65 ± 18.28*	9.26 ± 18.70	6.51	0.0892	0.076
FI	17.89 ± 26.02^ϯζ^	31.18 ± 29.04*^ζ^	43.25 ± 30.17*^ϯ^	50.62 ± 28.64*^ϯζ^	238.55	<0.001	0.367**

* Indicates *p* < 0.05 compared to no depression; ϯ indicates *p* < 0.05 compared to minor depression; ζ indicates *p* < 0.05 compared to moderate depression; * *indicates *p* < 0.001.

PF: Physical Functioning; RF: Role Functioning; CF: Cognitive Functioning; EF: Emotional Functioning; SF: Social Functioning; FA: Fatigue; NV: Nausea/Vomiting; PA: Pain; DY: Dyspnea; AP: Appetite; SL: Sleeplessness; CO: Constipation; DI: Diarrhea; FI: Financial hardship; QL: General health status.

### 3.3 Network structure and centrality

**Figure 1:**
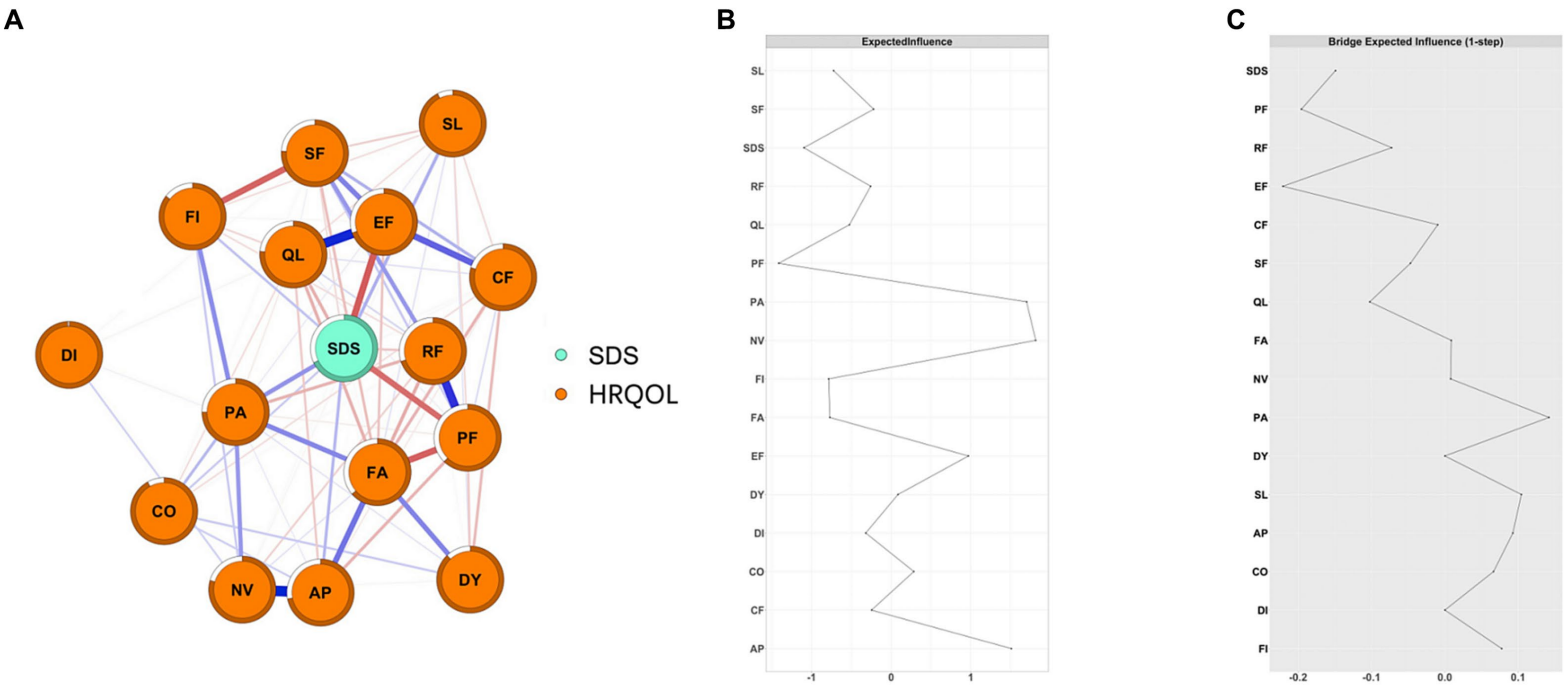
The network structure of depression and HRQOL in cancer patients and the EIs and BEIs of the nodes in the network. **(A)** network structure of depression and HRQOL in cancer patients; The blue edge represents positive connections and the red edge represents negative connections; the thickness and saturation of the edge represent the strength of the connection; **(B)** the Centrality plot depicting the Expected Influence of each node in the network; **(C)** the Bridge Centrality plot depicting the Bridge Expected Influence of each node in the networkPF: Physical Functioning; RF: Role Functioning; *CF*: Cognitive Functioning; EF: Emotional Functioning; SF: Social Functioning; FA: Fatigue; NV: Nausea/Vomiting; PA: Pain; DY: Dyspnea; AP: Appetite; SL: Sleeplessness; CO: Constipation; DI: Diarrhea; FI: Financial hardship; QL: General health status).

As shown in Figure 1A, we constructed a network of 15 HRQOL symptoms/functions and depression and found that depression was negatively connected with EF (weight = −0.22), PF (weight = −0.20), QL (weight = −0.11), RF (weight = −0.08), and SF (weight = −0.07) and positively connected with PA (weight = 0.16), SL (weight = 0.10), AP (weight = 0.09), FI (weight = 0.08), and CO (weight = 0.07) (Supplementary Table S2).

**Figure 2:**
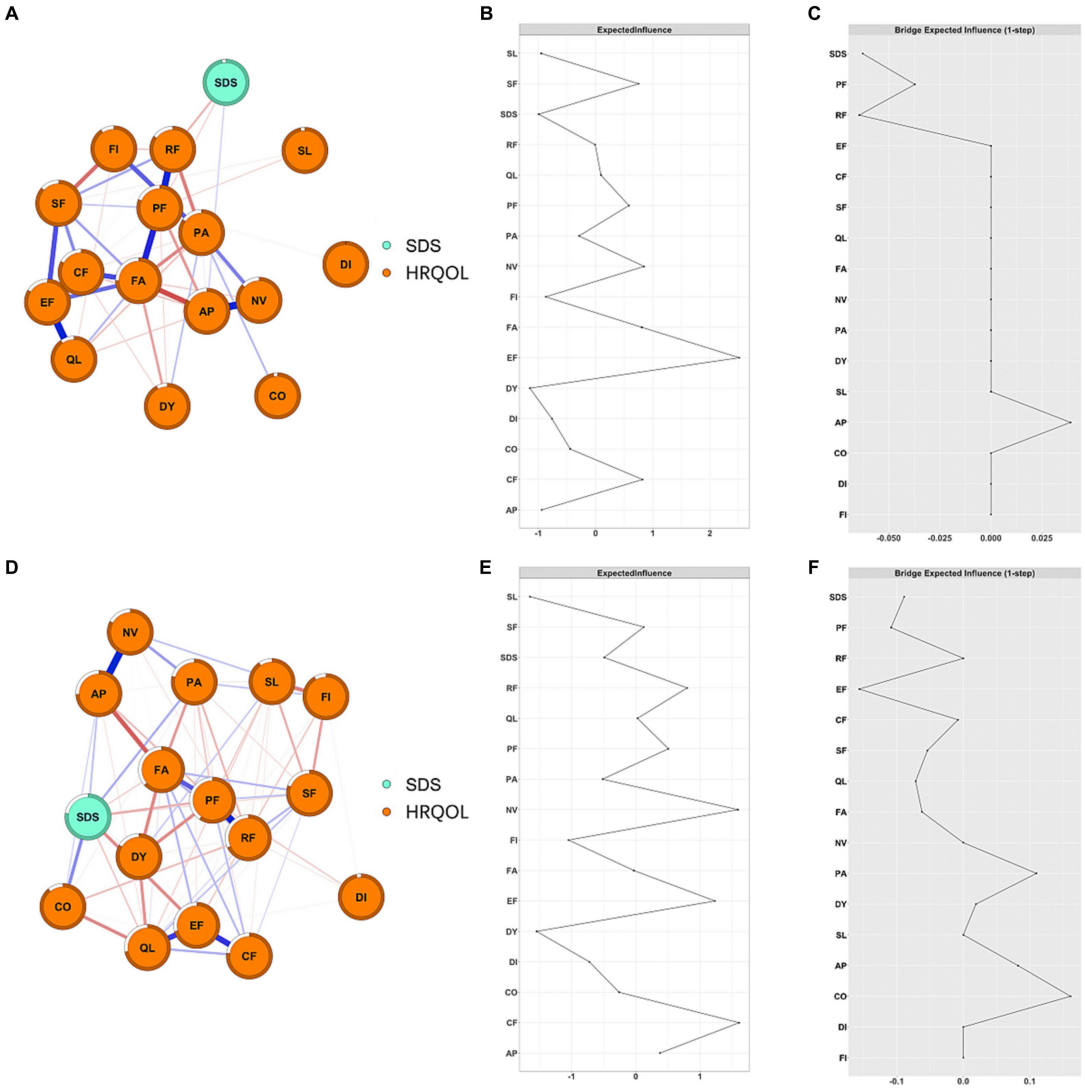
Network structure and EI of depression and HRQOL in cancer patients among different severities of depression. **(A)** Network structure of mild depression patients; (**B)** EI of mild depression patients; **(C)** bEI of mild depression patients; **(D)** network structure of moderate to severe depression patients; **(E)** EI of moderate to severe depression patients; **(F)** bEI of moderate to severe depression patients.

As shown in Figure 1B, NV and PA had the highest positive EI values (1.81 and 1.70, respectively), while SDS and PF had the highest negative EI values (−1.10 and − 1.41, respectively). EF, PF, PA, and SL were bridge symptoms/functions connecting depression and HRQOL dimensions, with bEI of −0.22, −0.20, 0.16, and 0.10, respectively (Figure 1C). Additionally, we also constructed depression and HRQOL dimensions networks among patients with different severity of depression and seven types of cancer, separately (Supplementary Figures S15–S20). NV was consistently the central symptom in patients with mild depression (EI = 1.76), moderate-to-severe (EI = 1.92), and no depression (EI = 1.30). PF was consistently the bridge function connecting depression and HRQOL dimensions in patients with mild and moderate-to-severe depression (Figure 2). Depression was the central symptom in all specific cancer types except gastric cancer. EF, PA, and PF were bridge symptoms connecting depression and HRQOL dimensions in most cancer types.

### 3.4 Network stability

The case-dropping bootstrap procedure showed that EI and bEI remained stable after dropping different proportions of the sample (Supplementary Figure S1). The CS-C for EI and bEI was 0.75. The bootstrapped 95% CIs for estimated edge weights indicated that most edges were stable and accurate (Supplementary Figures S2–S5).

### 3.5 Network comparison test

The network comparison test showed that the overall network structure was significantly different between patients aged ≥65 years and those aged <65 years (M = 0.215, *p* = 0.006), while the difference in global strength was not significant (S = 0.447, *p* = 0.181). We further analyzed the specific edges whose strengths were different between age groups and found that the edge EF-FA was significantly stronger in patients aged ≥65 years than in patients aged <65 (edge difference: 0.22; *p* < 0.001), and other edges such as EF-*CF* (difference: 0.16; *p* = 0.007) and EF-SF (difference: 0.13; *p* = 0.019) were significantly stronger in patients aged <65. No difference was observed between edges involving depression in all age groups (all *p* > 0.05) (Supplementary Figures S6–S14).

Both network structure (M = 0.22, *p* = 0.003) and global strength (S = 2.54, *p* < 0.001) were significantly different between the mild depression group and the moderate-to-severe depression group (Figure 2). Further analysis showed that the edges between SDS-EF (difference: 0.16; *p* < 0.001), between SDS-*CF* (difference: 0.16; *p* < 0.001), and between SL-FI (difference: 0.18; *p* < 0.001) were significantly stronger in the moderate-to-severe depression group compared to that in the mild depression group. The global strength showed that the network constructed in patients with moderate-to-severe depression was more densely connected compared to that in patients with mild depression.

Neither network structures nor global strengths were significantly different between TNM stages, genders, treatment types (surgery, chemotherapy, and/or radiotherapy), and months from first diagnosis (Supplementary Figures S6–S13).

### 3.6 Directed acyclic graph (DAG)

**Figure 3:**
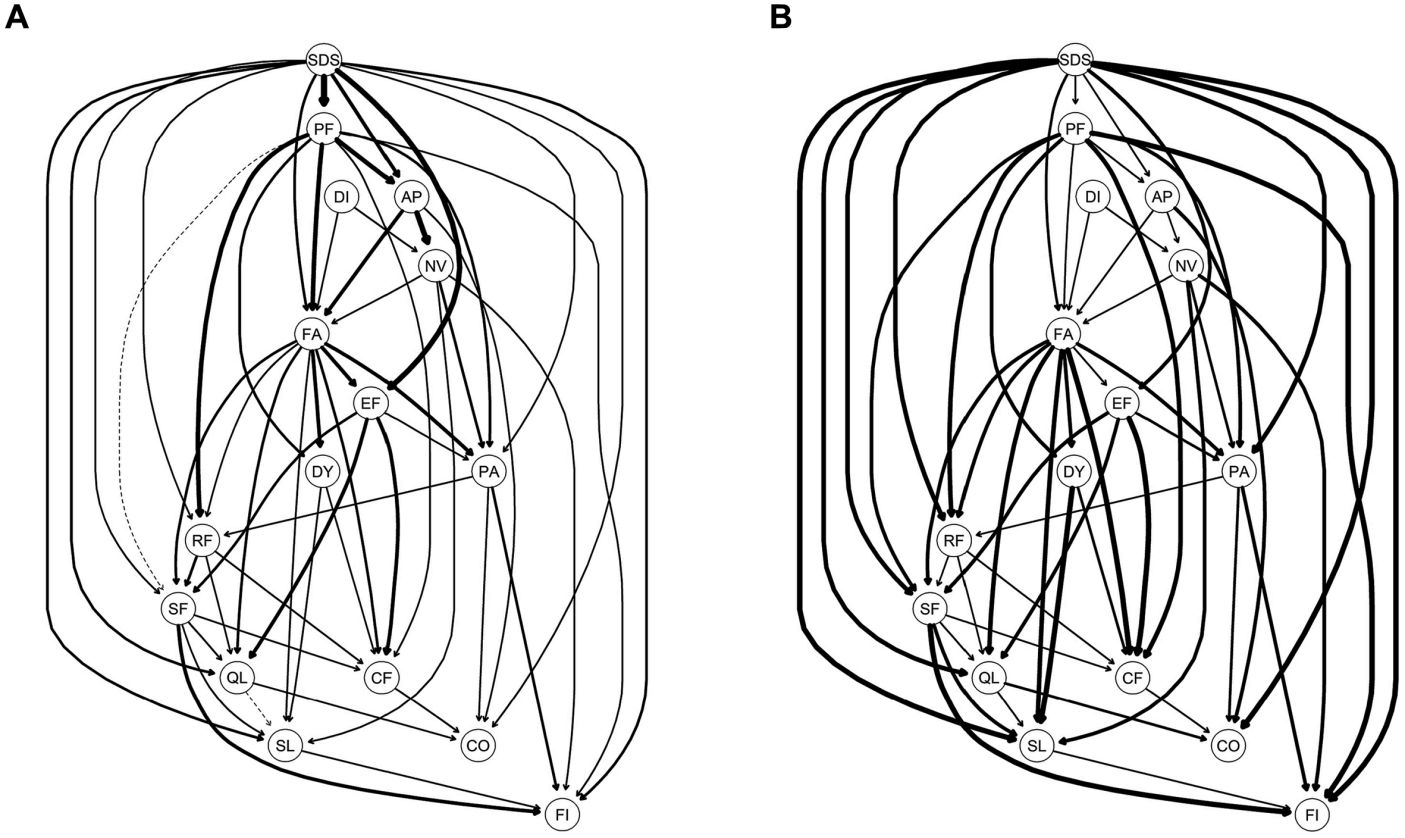
Directed acyclic graph (DAG) for symptoms of Quality of Life and depression; **(A)** the edge thickness represents the importance of that edge to the overall DAG structure; **(B)** the edge thickness represents the directional probability. **A**: Presence of edges: Edge thickness indicates the importance of that edge to the overall network structure, with greater thickness signifying that an edge is more crucial to the model fit. Thickness reflects the change in the Bayesian Information Criterion of the model when that edge is removed. For this graph, solid lines represent that the presence of an edge improves the model fit (a dashed line would represent an edge whose presence worsens the model fit). **B**: Direction of edges: the edge thickness indicates directional probability, or in what percentage of the fitted networks the edge went in that direction. Edge thickness is drawn proportionately such that a thicker arrow indicates a higher directional probability. For this graph, a solid line represents that an edge was present in its current direction in at least 51% of the 10,000 bootstrapped networks, while a dotted line represents an edge present in its current direction in less than 51%. For both **A,B**, exact edge weights can be found in Supplementary Table S1 in the supplementary materials.

Figure 3A displays the importance of each edge to the entire DAG structure. The edges that were most important for the network structure included SDS-PF (with a change in BIC of −373.95), AP-NV (with a change in BIC of −330.54), and SDS-EF (with a change in BIC of −155.93). Meanwhile, the edges that were least important for the network structure included SL-QL (with a change in BIC of 0.07) and SF-QL (with a BIC change of −0.54) (Supplementary Table S3).

In Figure 3B, an edge is thicker if it points from one node to another in a greater proportion of the bootstrapped networks. Structurally, SDS was at the top of the DAG, which directly activated a total of 11 symptoms/functions of HRQOL, including PF (BIC: −373.95; Direction: 0.60), EF (BIC: −155.93; Direction: 0.76), AP (BIC: −45.47; Direction: 0.58), FA (BIC: −31.49; Direction: 0.69), QL (BIC: −19.99; Direction: 0.94), SL (BIC: −18.68; Direction: 0.97), FI (BIC: −13.22; Direction: 0.99), PA (BIC: −12.78; Direction: 0.94), RF (BIC: −8.19; Direction: 0.92), CO (BIC: −5.79; Direction: 0.97), and SF (BIC: −5.77; Direction: 0.93) (Supplementary Table S3).

**Figure 4:**
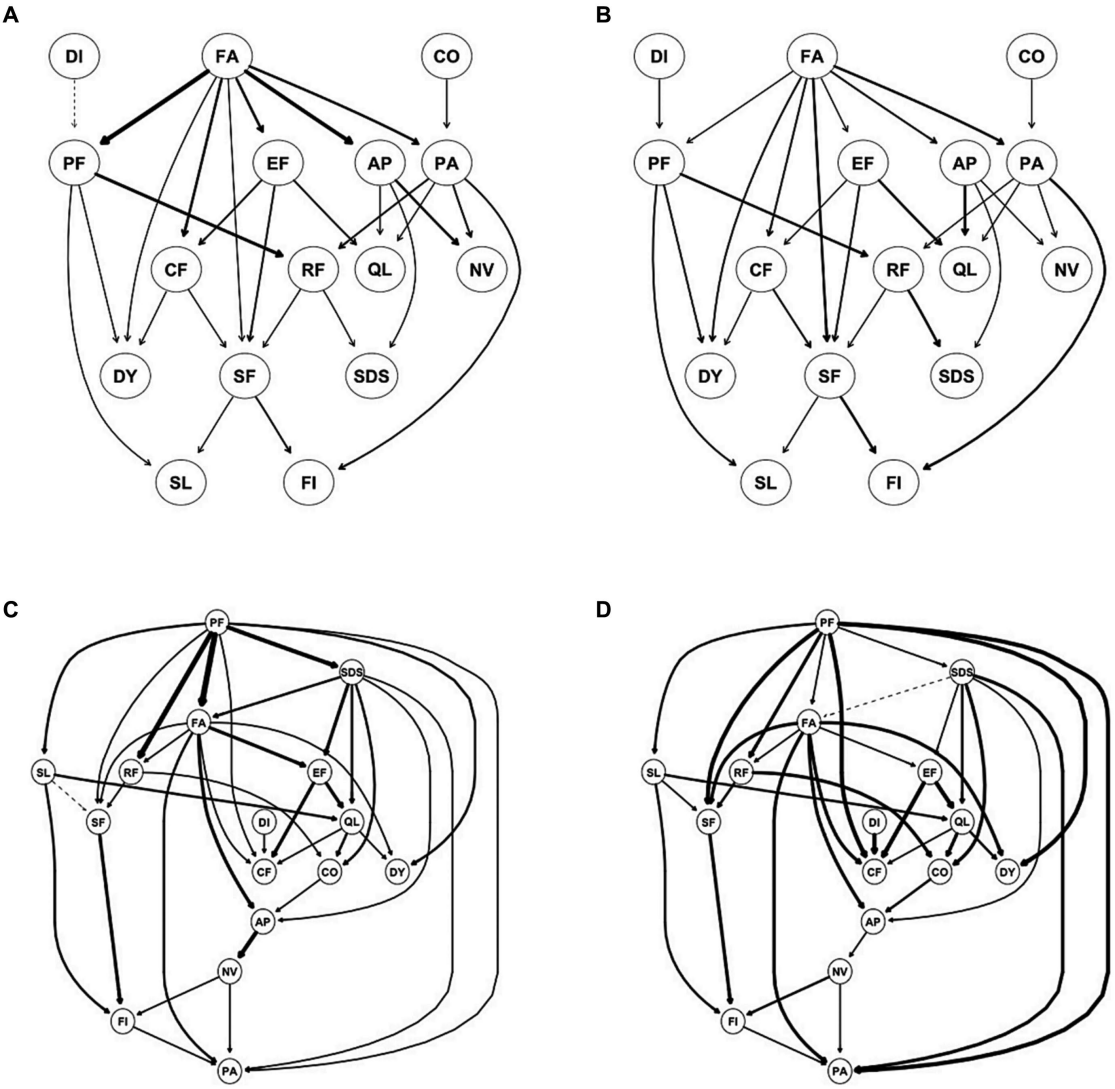
Directed acyclic graph (DAG) for symptoms/dimensions of HQOL and depression for mild depression **(A,B)** and moderate to severe depression **(C,D)**. **(A,C)** Edge thickness represents the importance of that edge to the overall DAG structure; **(B,D)** edge thickness represents the directional probability.

Using DAG analysis, we further analyzed the potential causal relationship between depression and dimensions of HRQOL in patients with mild depression and moderate-to-severe depression, separately. In patients with mild depression, SDS was downstream of DAG and indirectly activated by FA through AP, RF, and PA. In patients with moderate-to-severe depression, SDS was upstream of DAG and directly activated PA and FA but indirectly activated RF and AP through FA (Figure 4). In patients with no depression, FA was upstream of DAG and directly activated PA, QL, NV, AP, RF, SF, DY, and SL (Supplementary Figure S14).

## 4 Discussion

To the best of our knowledge, this is the first study to utilize network analysis to investigate the interrelationship between different dimensions of HRQOL and depression among cancer patients. In this study, we constructed a depression and HRQOL dimensions network in heterogeneous cancer patients. Our study results showed that depression and 15 dimensions of the EORTC-QLQ-C30 questionnaire were highly intercorrelated and could be represented as a network, indicating the validity of the EORTC-QLQ-C30 questionnaire. Cancer patients may experience symptoms, such as pain, nausea, vomiting, fatigue, loss of appetite, depression, anxiety, constipation, shortness of breath, insomnia, etc. due to cancer itself, its treatment, and co-morbid conditions (38, 39). In this study, we found that NV, depression, and PA were the central symptoms in the network while the PF was the central function, and they remained central in patients with specific types of cancer. In the network structure, nodes with high EI acted as important intermediaries or connectors within the network, facilitating the flow of information, spreading influence, or affecting the overall network structure and function (30). Therefore, NV, depression, PA, and PF may play a crucial role in determining overall HRQOL in cancer patients, which might be important targets for clinical intervention to improve overall HRQOL.

In our study, 68% of cancer patients had depression, and nearly half of them were moderate-to-severe depression. Furthermore, our study showed that the severity of depression was associated with almost all dimensions (symptoms/functions) of HRQOL in cancer patients except diarrhea; this was similar to results in previous studies (9, 40, 41). However, previous studies did not account for complex interactions between symptoms/functions, and the potential causal association between symptoms/functions was not investigated. In this study, we used EORTC QLQ-C30 questionnaire to assess HRQOL of cancer patients, and we found that depression was the central symptom in HRQOL-depression networks, and EF, PF, PA, and SL were bridge symptoms/functions linking depression and HRQOL dimensions. Additionally, DAG analysis showed that depression was at the top of DAG and directly activated 11 symptoms/functions of HRQOL dimensions, particularly EF, PF, and PA, which further triggered other symptoms/functions of HRQOL. Our study illustrated the direction and pathway of the impact of depression on HRQOL for the first time. These results suggested that screening and early treatment of depression in cancer patients was vital to improve the mental and physical health of cancer patients and improve overall HRQOL.

In this study, we found that the relationship between depression and HRQOL dimensions might be bidirectional. In stratified analysis, depression was downstream of DAG and was mainly activated by RF and AP in patients with mild depression, while depression was upstream of DAG in patients with severe depression, which influenced other symptoms and functions of HRQOL. Recent studies indicated that PA might worsen depressive symptoms in cancer patients (42–44), while treatment of depression might facilitate effective management of pain in cancer patients (45). This indicated that the relationship between PA and depression might be bidirectional. However, this result should be interpreted with caution. Although we used DAG analysis to show the potential direction between symptoms/functions, still the study design we applied was cross-sectional. Therefore, further longitudinal studies are needed to establish a more definitive understanding of the causal dynamics between depression and pain.

## 5 Limitation

There were several limitations in this study. First, the data we used were all derived from a cross-sectional survey; therefore, the dynamic changes between depression and HRQOL dimensions could not be examined. Second, although we used the DAG network to explore the predictive (and potentially causal) priority of these symptoms, we were unable to establish a definitive causal relationship due to the constraints imposed by our cross-sectional design. Third, the results should be interpreted with caution as the generated networks were based on group-level analysis, and whether group-level results can represent individuals remained unclear. Finally, our study was conducted in a single medical center, which might have selection bias.

## 6 Conclusion

Our study explored inter-connection, bridge symptoms, and potential causal relationships between depression and different dimensions of HRQOL in Chinese cancer patients. We found that nearly two-thirds of cancer patients experienced various degrees of depression, and depression was the central symptom in the depression-HRQOL dimensions network, in addition to NV, PA, and PF. DAG and bridge analysis indicated that depression might influence overall HRQOL in cancer patients through EF, PA, PF, and SL, particularly in patients with moderate-to-severe depression. The disparity in network structures between mild and moderate-to-severe depression suggested that the relationship between depression and HRQOL dimensions might be bidirectional. Screening and early treatment of depression were warranted to improve the overall HRQOL of cancer patients, in addition to adequate treatment of PA and NV and improvement in PF.

## Data availability statement

The raw data supporting the conclusions of this article will be made available by the authors, without undue reservation.

## Ethics statement

The studies involving humans were approved by the study was approved by Xinjiang Medical University ethnic committee (Number: XJYKDXR20230208001). All sensitive and private information of patients were kept confidential. All participants provided written consent.

## Author contributions

SM: Data curation, Formal analysis, Investigation, Project administration, Software, Supervision, Validation, Writing – original draft, Writing – review & editing. PM: Conceptualization, Data curation, Funding acquisition, Investigation, Methodology, Software, Supervision, Writing – original draft, Writing – review & editing. KA: Funding acquisition, Project administration, Resources, Visualization, Writing – review & editing HZ: Data curation, Formal analysis, Methodology, Writing – review & editing. WT: Conceptualization, Data curation, Methodology, Writing – review & editing. KT: Data curation, Methodology, Writing – review & editing. YL: Data curation, Methodology, Writing – review & editing. QS: Data curation, Methodology, Writing – review & editing. WJ: Conceptualization, Investigation, Software, Supervision, Writing – original draft, Writing – review & editing.
