# Supplementary material for: A network approach to investigating the inter-relationship between health-related quality of life dimensions and depression in 1735 Chinese patients with heterogeneous cancers
Source: Front Public Health. 2024 Jan 23;11:1325986. doi: 10.3389/fpubh.2023.1325986 (PMC10844480; doi:10.3389/fpubh.2023.1325986)
Supplement: Supplementary file 1 [file Data_Sheet_1.DOCX]

**Supplementary Material**

**Table S1 Prevalence of depression and eight common EORTC-QLQ-C30 symptoms by cancer types;**

**Table S2 Partial correlation matrix between depression and HRQOL in cancer patients**

**Table S3 Edges in directed acyclic graphs**

**Figure S1 Stability of node expected influences and the bridge expected influencein the network**

**Figure S2 Confidence intervals for edge weights**

**Figure S3 Non-parametric bootstrap edge difference test**

**Figure S4 Non-parametric bootstrap EI difference test**

**Figure S5 Non-parametric bootstrap bridgeEI difference test**

**Figure S6 Comparison of network structure and centrality between males and females**

**Figure S7 Comparison of network structure and centrality between different age groups**

**Figure S8 Comparison of network structure and centrality between different TNM stage**

**Figure S9 Comparison of network structure and centrality between whether surgery patients**

**Figure S10 Comparison of network structure and centrality between whether chemotherapy patients**

**Figure S11 Comparison of network structure and centrality between whether radiotherapy patients**

**Figure S12 Comparison of network structure and centrality between patients which month from diagnosis<12Month and month from diagnosis≥12Month**

**Figure S13 Network structure and expected influence for HRQOL dimensions of no depression patients:**

**Figure S14 Directed acyclic graph (DAG) for HRQOL dimensions of no depression patients**

**Figure S15 Estimation network structure of the relationship between depression and HRQOL in breast cancer**

**Figure S16 Estimation network structure of the relationship between depression and HRQOL in Colorectal cancer**

**Figure S17 Estimation network structure of the relationship between depression and HRQOL in cervical cancer**

**Figure S18 Estimation network structure of the relationship between depression and HRQOL in gastric cancer**

**Figure S19 Estimation network structure of the relationship between depression and HRQOL in head and neck cancer**

**Figure S20 Estimation network structure of the relationship between depression and HRQOL in esophagus cancer**

**Table S1.**

**Prevalence of depression and eight common EORTC-QLQ-C30 symptoms by cancer types (n,%)**

| **Symptom** | **total (n=1735)** | **Breast (n=462)** | **Colorectal (n=297)** | **Cervical (n=287)** | **Gastric (n=217)** | **Head and neck* (n=191)** | **Esophagus (n=189)** | **Lung (n=92)** |
| --- | --- | --- | --- | --- | --- | --- | --- | --- |
| **Depression** | 1167(67.26) | 245(53.03) | 171(57.58) | 231(80.49) | 156(71.89) | 124(64.92) | 166(87.83) | 74(80.43) |
| **FA** | 1398(80.58) | 328(71.00) | 230(77.44) | 255(88.85) | 187(86.18) | 150(78.53) | 167(88.36) | 81(88.04) |
| **NV** | 411(23.69) | 76(16.45) | 58(19.53) | 94(32.75) | 73(33.64) | 35(18.32) | 54(28.57) | 21(22.83) |
| **PA** | 898(51.76) | 189(40.91) | 121(40.74) | 200(69.69) | 111(51.15) | 95(49.74) | 128(67.72) | 54(58.70) |
| **DY** | 579(33.37) | 141(30.52) | 90(30.30) | 83(28.92) | 76(35.02) | 61(31.94) | 69(36.51) | 59(64.13) |
| **SL** | 1141(65.76) | 307(66.45) | 188(63.3) | 171(59.58) | 147(67.74) | 130(68.06) | 134(70.9) | 64(69.57) |
| **AP** | 594(34.24) | 97(21.00) | 89(29.97) | 124(43.21) | 109(50.23) | 50(26.18) | 88(46.56) | 37(40.22) |
| **CO** | 630(36.31) | 136(29.44) | 104(35.02) | 111(38.68) | 104(47.93) | 63(32.98) | 82(43.39) | 30(32.61) |
| **DI** | 306(17.64) | 44(9.52) | 76(25.59) | 71(24.74) | 48(22.12) | 30(15.71) | 28(14.81) | 9(9.78) |

**PF: Physical Functioning; RF: Role Functioning; CF: Cognitive Functioning; EF: Emotional Functioning; SF: Social Functioning; FA: Fatigue; NV: Nausea/Vomiting; PA: Pain; DY: Dyspnea; AP: Appetite; SL:** S**leeplessness; CO: Constipation; DI: Diarrhoea; FI: Financial hardship; QL: General health status**

**Head and Neck*: Nasal cancer; Laryngeal cancer; Thyroid cancer; Oral cancer; Tongue cancer; Lip cancer**

**Table S2**

**Partial correlation matrix between depression and HRQOL in cancer patients**

|  | **SDS** | **PF** | **RF** | **EF** | **CF** | **SF** | **QL** | **FA** | **NV** | **PA** | **DY** | **SL** | **AP** | **CO** | **DI** | **FI** |
| --- | --- | --- | --- | --- | --- | --- | --- | --- | --- | --- | --- | --- | --- | --- | --- | --- |
| **SDS** | 0.00 | -0.20 | -0.07 | -0.22 | -0.01 | -0.05 | -0.10 | 0.01 | 0.01 | 0.14 | 0.00 | 0.10 | 0.09 | 0.07 | 0.00 | 0.08 |
| **PF** | -0.20 | 0.00 | 0.28 | 0.00 | 0.05 | 0.07 | 0.02 | -0.21 | 0.00 | -0.03 | -0.07 | -0.05 | -0.10 | 0.00 | 0.00 | 0.00 |
| **RF** | -0.07 | 0.28 | 0.00 | 0.00 | 0.00 | 0.14 | 0.05 | -0.11 | -0.06 | -0.10 | -0.01 | 0.00 | 0.00 | -0.01 | 0.00 | -0.05 |
| **EF** | -0.22 | 0.00 | 0.00 | 0.00 | 0.21 | 0.17 | 0.32 | -0.08 | 0.00 | 0.00 | 0.00 | 0.00 | 0.00 | 0.00 | 0.00 | -0.01 |
| **CF** | -0.01 | 0.05 | 0.00 | 0.21 | 0.00 | 0.10 | 0.04 | -0.10 | 0.00 | -0.01 | -0.09 | -0.05 | -0.03 | -0.04 | 0.00 | 0.00 |
| **SF** | -0.05 | 0.07 | 0.14 | 0.17 | 0.10 | 0.00 | 0.02 | -0.08 | 0.00 | 0.00 | 0.00 | -0.06 | 0.00 | -0.01 | -0.01 | -0.21 |
| **QL** | -0.10 | 0.02 | 0.05 | 0.32 | 0.04 | 0.02 | 0.00 | -0.10 | 0.00 | -0.03 | 0.00 | -0.04 | -0.07 | -0.04 | -0.02 | -0.05 |
| **FA** | 0.01 | -0.21 | -0.11 | -0.08 | -0.10 | -0.08 | -0.10 | 0.00 | 0.04 | 0.16 | 0.16 | 0.04 | 0.19 | 0.00 | 0.02 | 0.00 |
| **NV** | 0.01 | 0.00 | -0.06 | 0.00 | 0.00 | 0.00 | 0.00 | 0.04 | 0.00 | 0.14 | 0.01 | -0.01 | 0.35 | 0.00 | 0.06 | 0.07 |
| **PA** | 0.14 | -0.03 | -0.10 | 0.00 | -0.01 | 0.00 | -0.03 | 0.16 | 0.14 | 0.00 | 0.04 | 0.00 | 0.03 | 0.09 | 0.00 | 0.15 |
| **DY** | 0.00 | -0.07 | -0.01 | 0.00 | -0.09 | 0.00 | 0.00 | 0.16 | 0.01 | 0.04 | 0.00 | 0.04 | 0.00 | 0.07 | 0.00 | 0.00 |
| **SL** | 0.10 | -0.05 | 0.00 | 0.00 | -0.05 | -0.06 | -0.04 | 0.04 | -0.01 | 0.00 | 0.04 | 0.00 | 0.00 | 0.03 | 0.00 | -0.04 |
| **AP** | 0.09 | -0.10 | 0.00 | 0.00 | -0.03 | 0.00 | -0.07 | 0.19 | 0.35 | 0.03 | 0.00 | 0.00 | 0.00 | 0.06 | 0.00 | 0.00 |
| **CO** | 0.07 | 0.00 | -0.01 | 0.00 | -0.04 | -0.01 | -0.04 | 0.00 | 0.00 | 0.09 | 0.07 | 0.03 | 0.06 | 0.00 | 0.00 | 0.00 |
| **DI** | 0.00 | 0.00 | 0.00 | 0.00 | 0.00 | -0.01 | -0.02 | 0.02 | 0.06 | 0.00 | 0.00 | 0.00 | 0.00 | 0.00 | 0.00 | 0.00 |
| **FI** | 0.08 | 0.00 | -0.05 | -0.01 | 0.00 | -0.21 | -0.05 | 0.00 | 0.07 | 0.15 | 0.00 | -0.04 | 0.00 | 0.00 | 0.00 | 0.00 |

**Table S3.**

**Edges in directed acyclic graphs**

| **From** | **To** | **BIC** | **Direction** |
| --- | --- | --- | --- |
| SDS | PF | -373.95 | 0.6 |
| AP | NV | -330.54 | 0.65 |
| SDS | EF | -155.93 | 0.76 |
| PF | RF | -150.54 | 0.91 |
| PF | FA | -132.44 | 0.58 |
| PF | AP | -91.37 | 0.52 |
| FA | EF | -87.94 | 0.62 |
| EF | CF | -79.37 | 0.96 |
| SF | FI | -74.27 | 0.95 |
| EF | QL | -66.8 | 0.81 |
| AP | FA | -48.63 | 0.58 |
| SDS | AP | -45.47 | 0.58 |
| FA | DY | -40 | 0.77 |
| FA | PA | -39.29 | 0.88 |
| PF | DY | -32.94 | 0.79 |
| EF | SF | -32.91 | 0.78 |
| SDS | FA | -31.49 | 0.69 |
| NV | PA | -28.82 | 0.7 |
| PA | FI | -26.72 | 0.86 |
| RF | SF | -20.14 | 0.6 |
| SDS | QL | -19.99 | 0.94 |
| SDS | SL | -18.68 | 0.97 |
| FA | SF | -16.69 | 0.9 |
| FA | CF | -16.63 | 0.96 |
| FA | QL | -16.28 | 0.91 |
| PF | PA | -14.02 | 0.89 |
| SDS | FI | -13.22 | 0.99 |
| SDS | PA | -12.78 | 0.94 |
| PA | RF | -12 | 0.61 |
| FA | RF | -10.69 | 0.89 |
| SF | CF | -8.72 | 0.57 |
| SDS | RF | -8.19 | 0.92 |
| DY | CF | -6.69 | 0.73 |
| PF | CF | -6.43 | 0.95 |
| EF | PA | -6.36 | 0.71 |
| PA | CO | -6.06 | 0.75 |
| SDS | CO | -5.79 | 0.97 |
| SDS | SF | -5.77 | 0.93 |
| AP | CO | -4.72 | 0.86 |
| NV | SL | -3.97 | 0.8 |
| SL | FI | -3.5 | 0.53 |
| RF | CF | -2.66 | 0.57 |
| NV | FI | -2.64 | 0.88 |
| RF | QL | -2.51 | 0.61 |
| DI | FA | -2.38 | 0.65 |
| DI | NV | -2.3 | 0.55 |
| SF | SL | -2.21 | 0.78 |
| NV | FA | -1.51 | 0.59 |
| SF | QL | -1.3 | 0.62 |
| PF | FI | -1.06 | 1 |
| DY | SL | -0.88 | 0.97 |
| QL | CO | -0.79 | 0.69 |
| CF | CO | -0.56 | 0.67 |
| FA | SL | -0.32 | 0.92 |
| QL | SL | 0.8 | 0.57 |
| PF | SF | 0.92 | 0.83 |

**Figure S1.**

**Stability of node expected influences and the bridge expected influencein the network.**

**
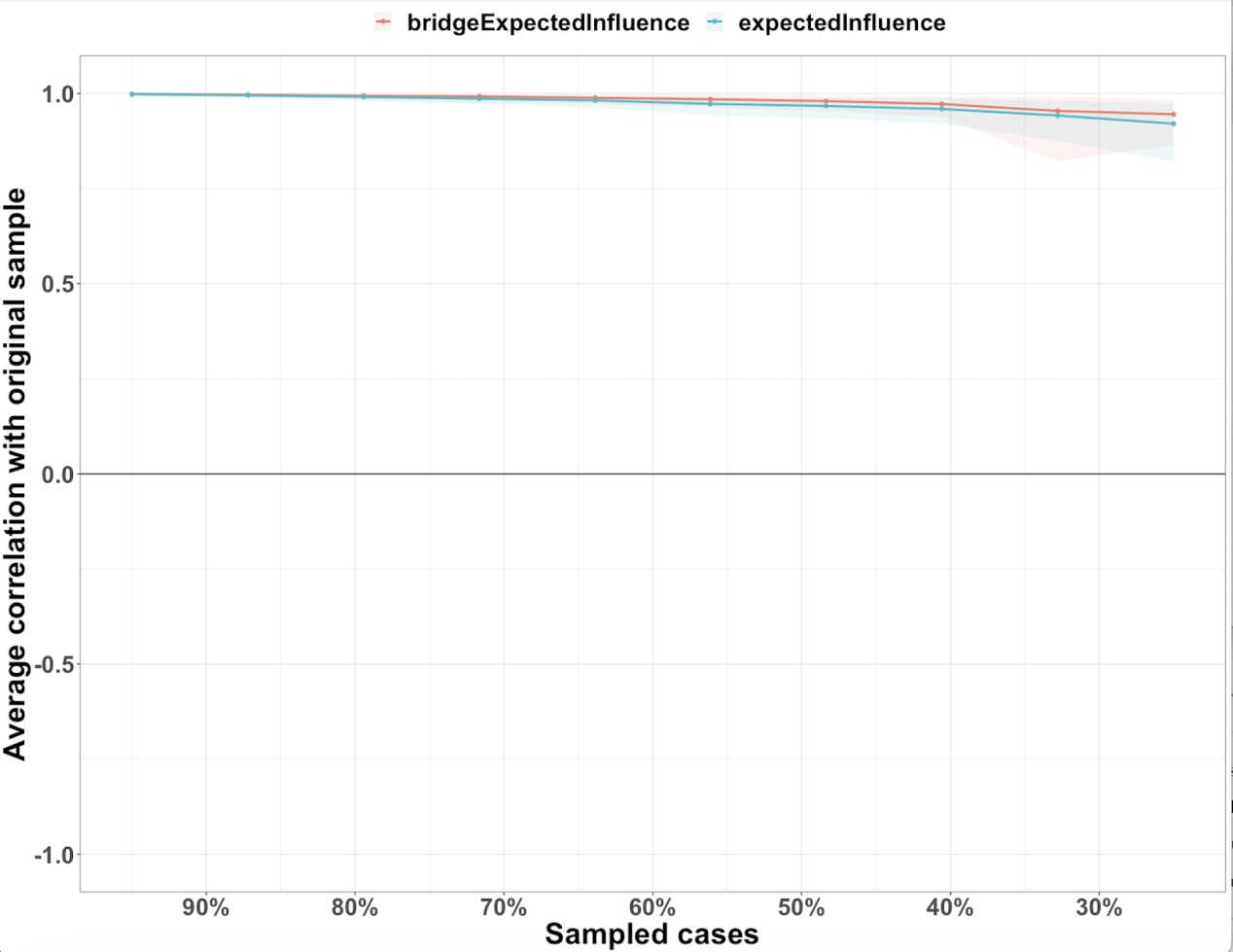
**

**Figure S2.**

**Confidence intervals for edge weights**

**
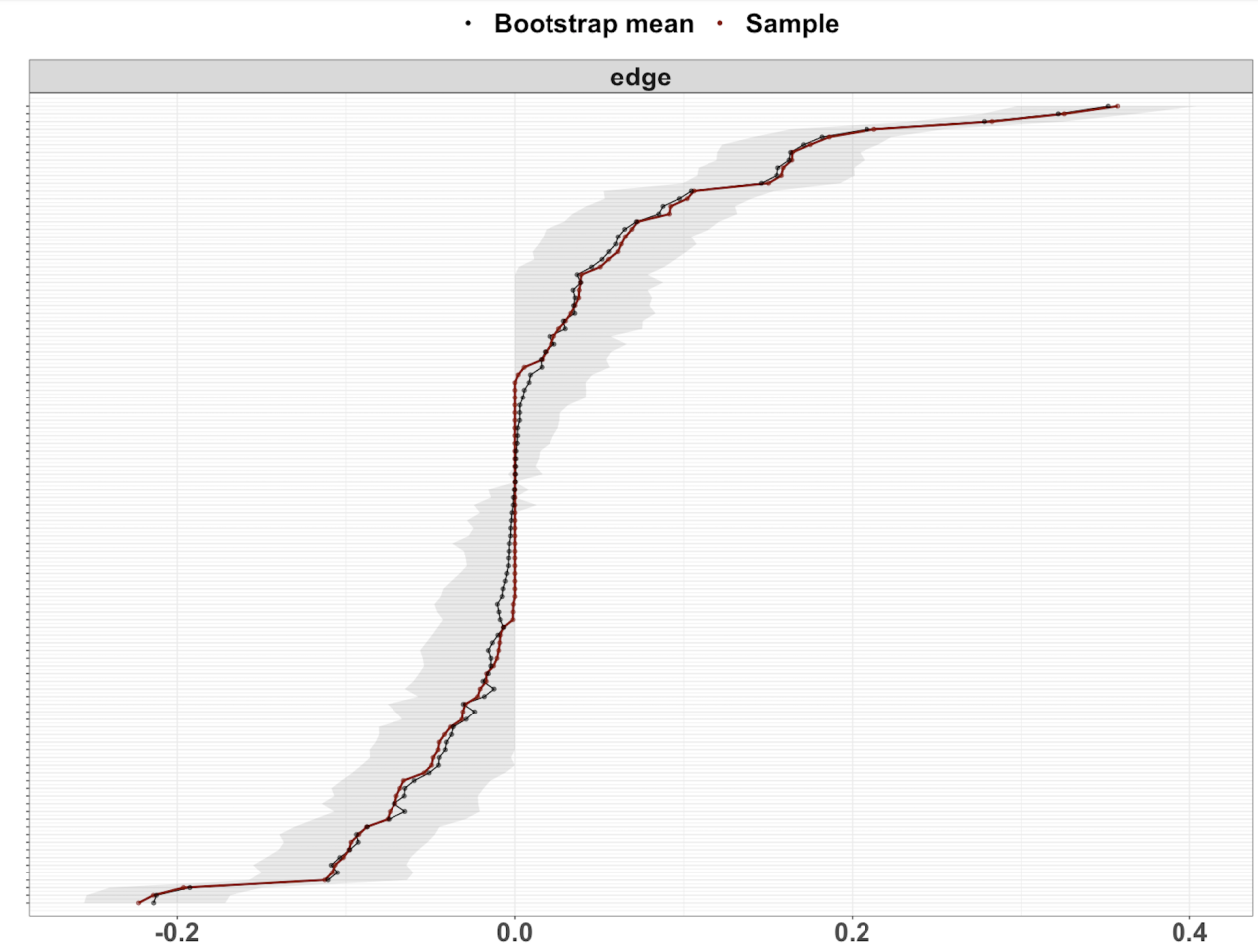
**

**Figure S3.**

**Non-parametric bootstrap edge difference test**

**
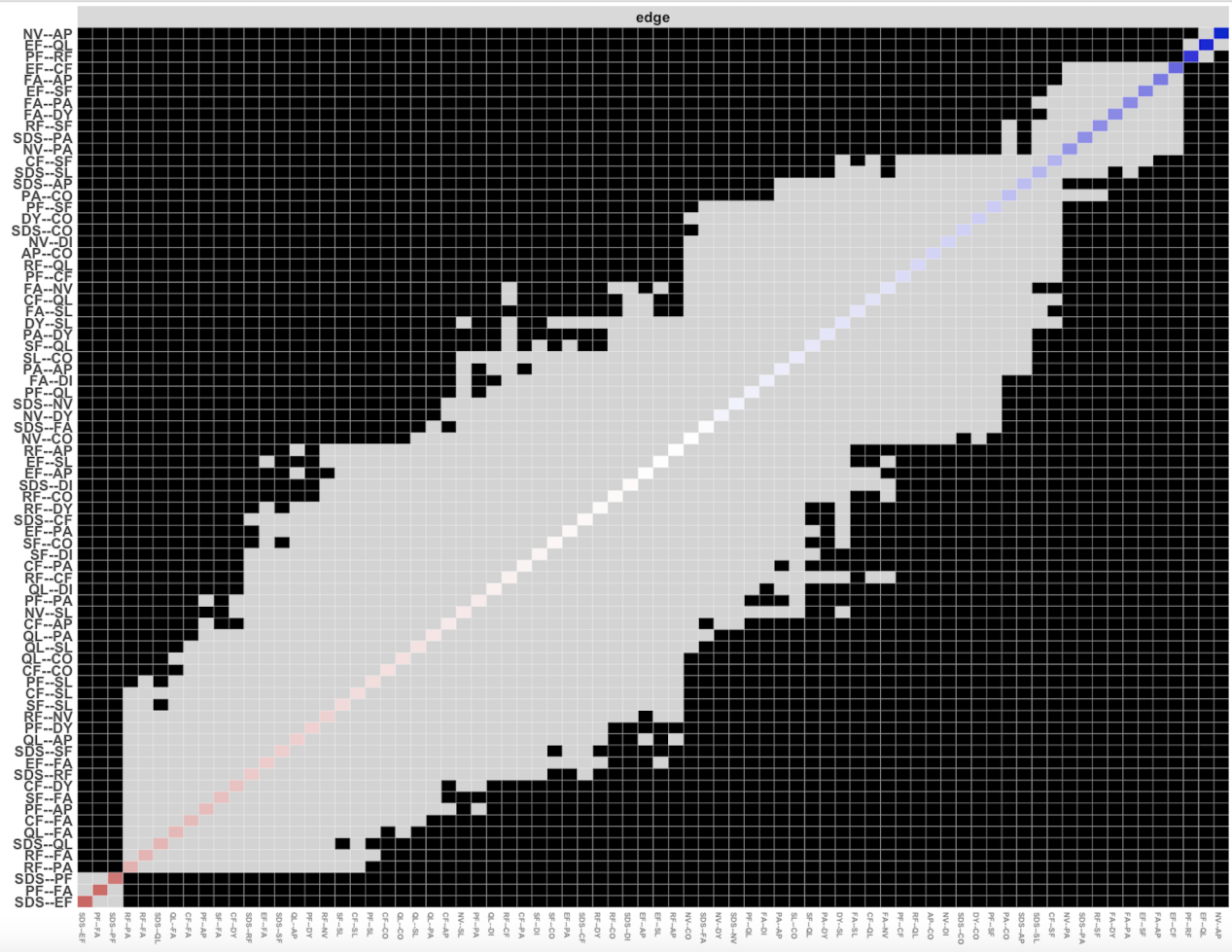
**

**Figure S4.**

**Non-parametric bootstrap EI difference test**


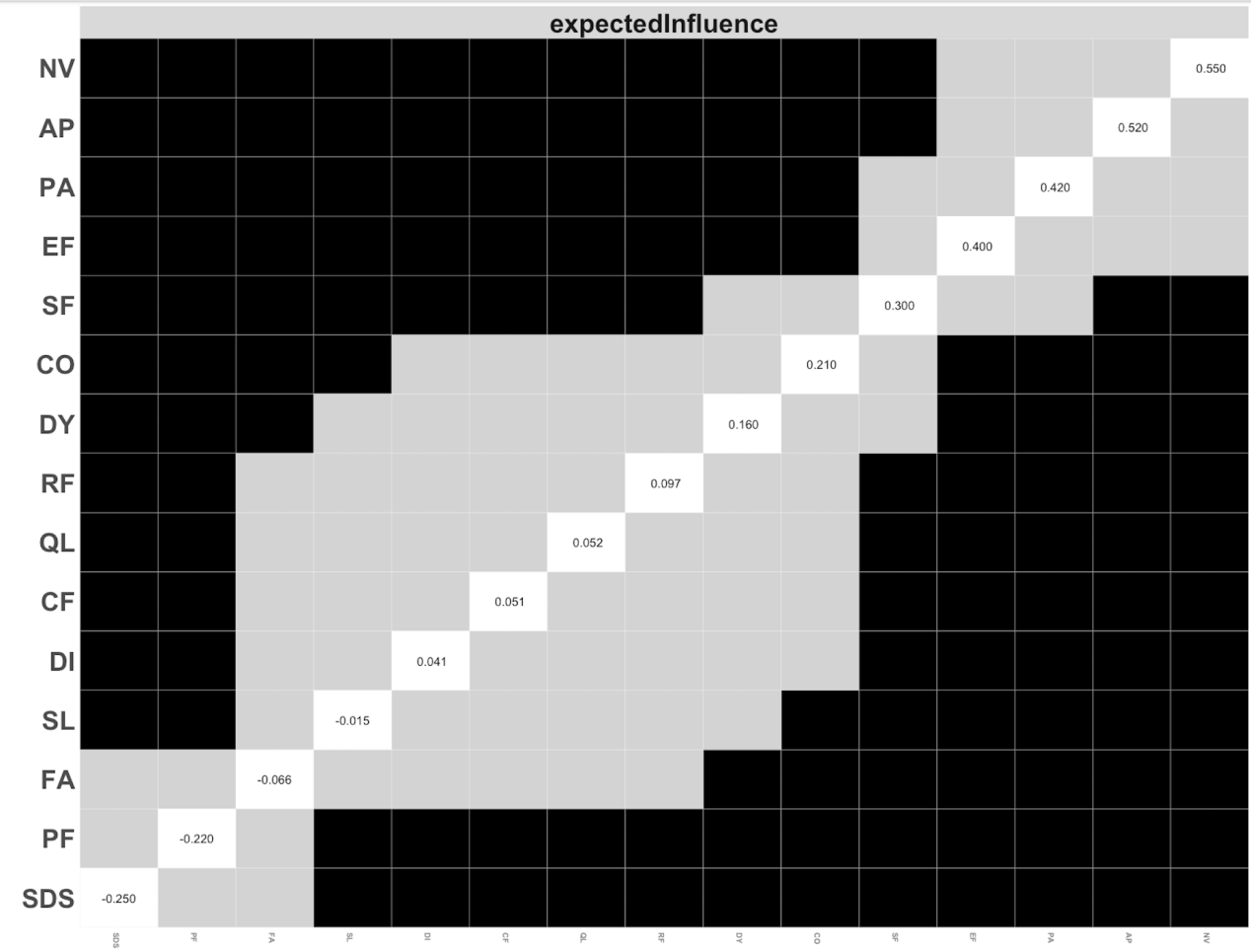


**Figure S5.**

**Non-parametric bootstrap bridgeEI difference test**

**
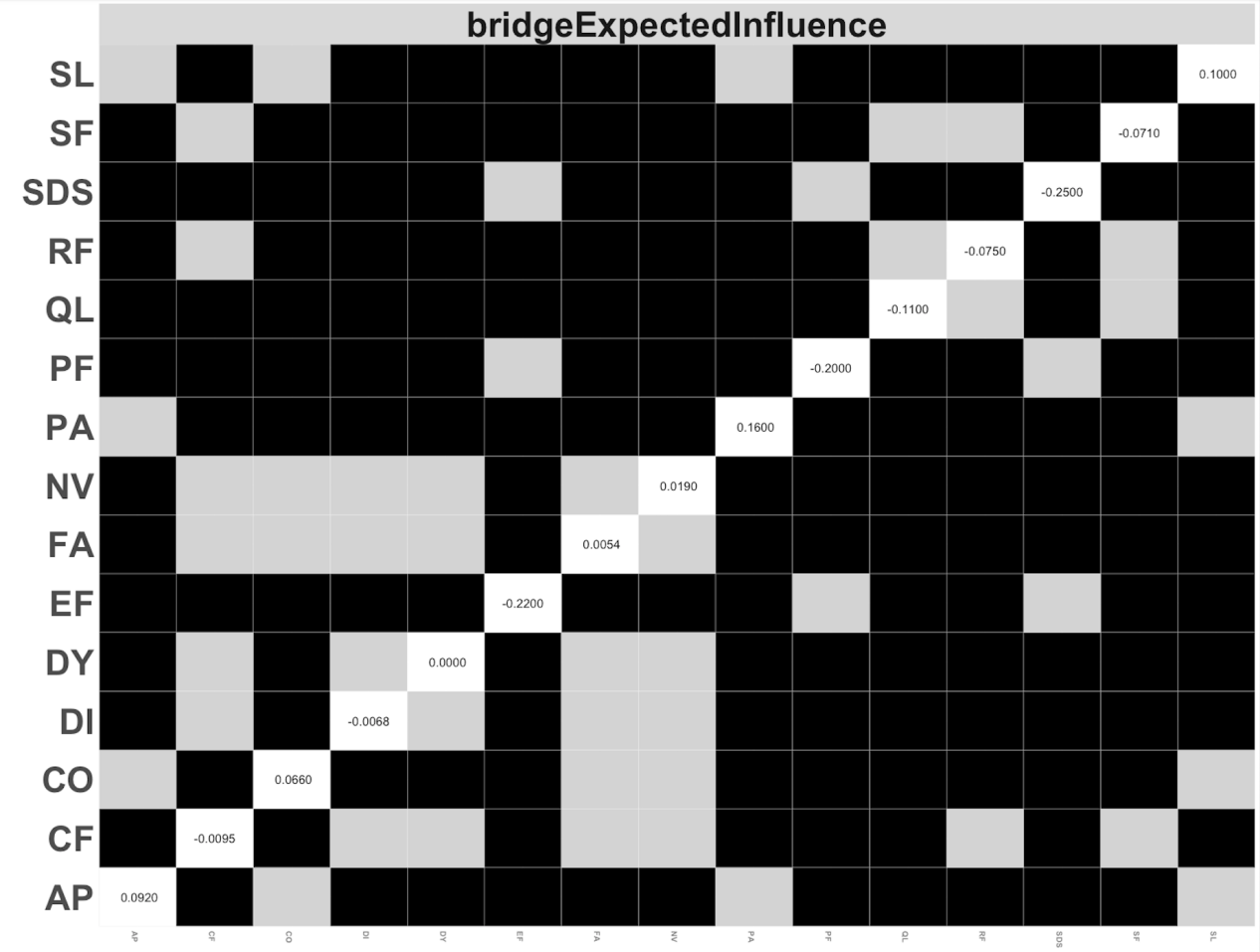
**

**Figure S6.**

**Comparison of network structure and centrality between males and females; (M = 0.141, p = 0.056); (S = 0.005, p = 0.98)**

**A Network structure; B Expected influnce; C Bridge Expected influnce: Males;**

**D Network structure; E Expected influnce; F Bridge Expected influnce: Females;**


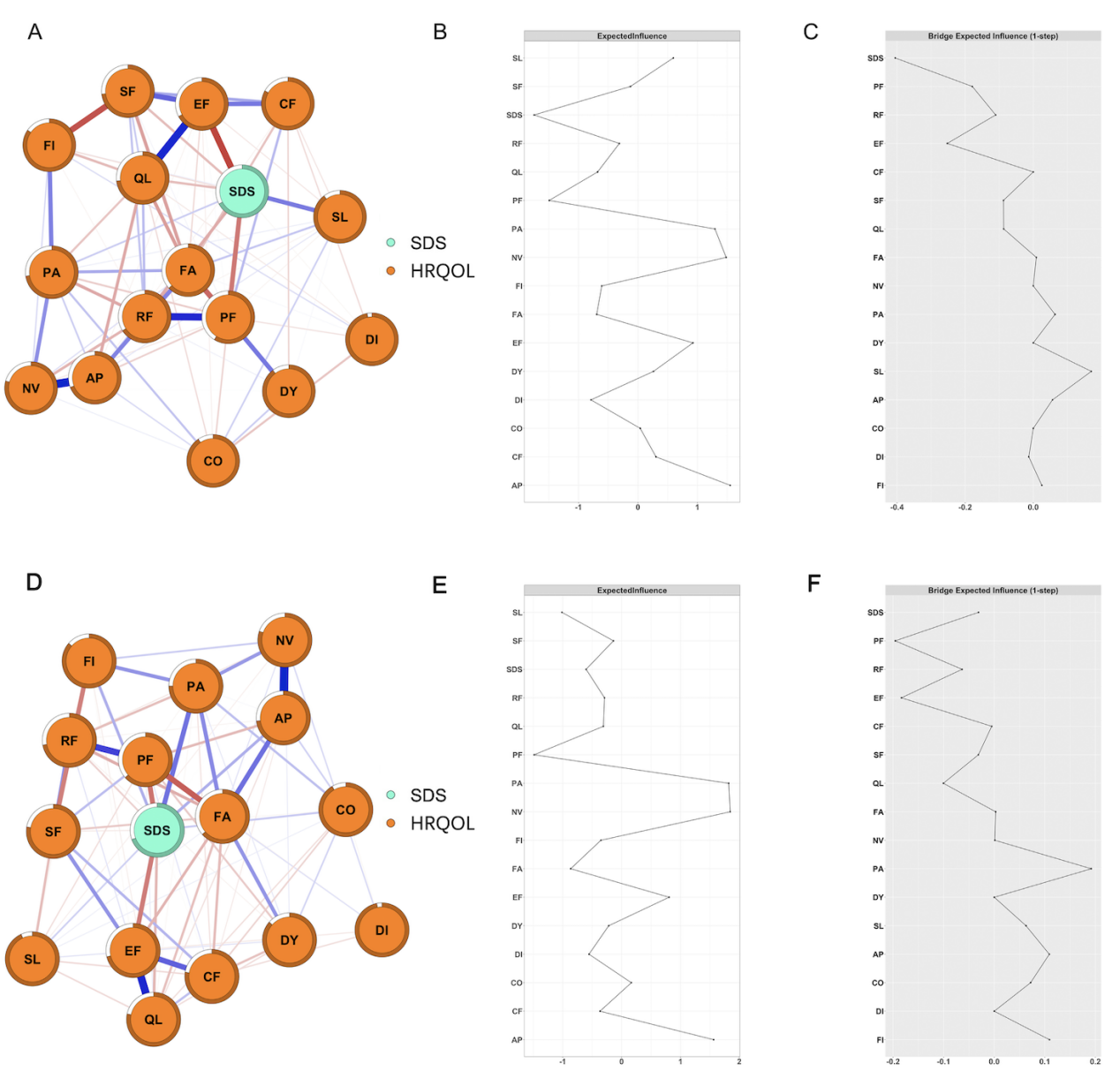


**Figure S7.**

**Comparison of network structure and centrality between different age groups; (M = 0.21, p = 0.006); (S = 0.447, p = 0.181)**

**A Network structure; B Expected influnce; C Bridge Expected influnce : Elderly groups(Age≥65);**

**D Network structure; E Expected influnce; F Bridge Expected influnce: General groups(Age<65);**

**
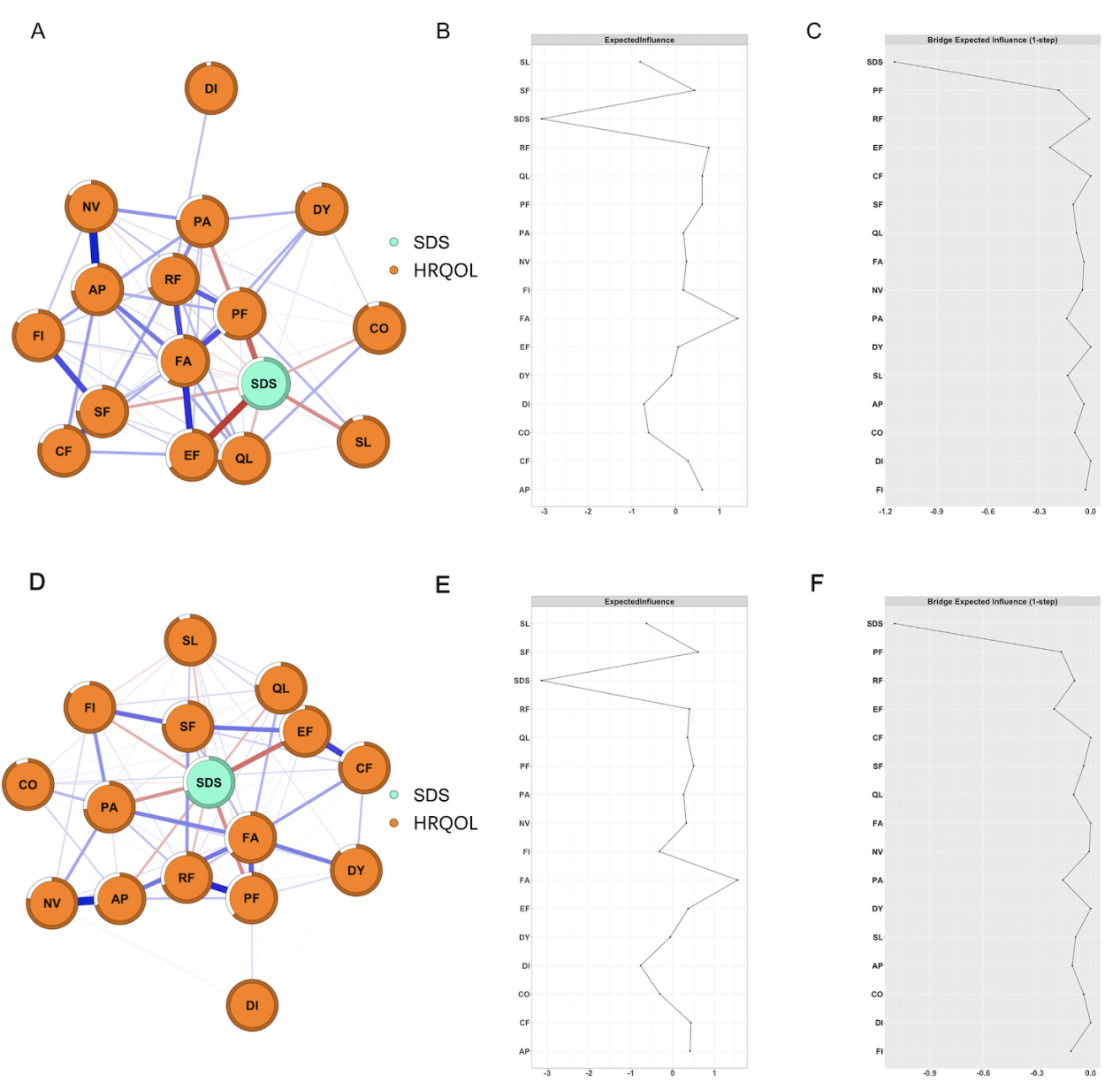
**

**Figure S8.**

**Comparison of network structure and centrality between different TNM stage; (M = 0.11, p = 0.48); (S = 0.005, p = 0.98)**

**A Network structure; B Expected influnce; C Bridge Expected influnce: Elderly groups(Age≥65);**

**D Network structure; E Expected influnce; F Bridge Expected influnce: General groups(Age<65);**


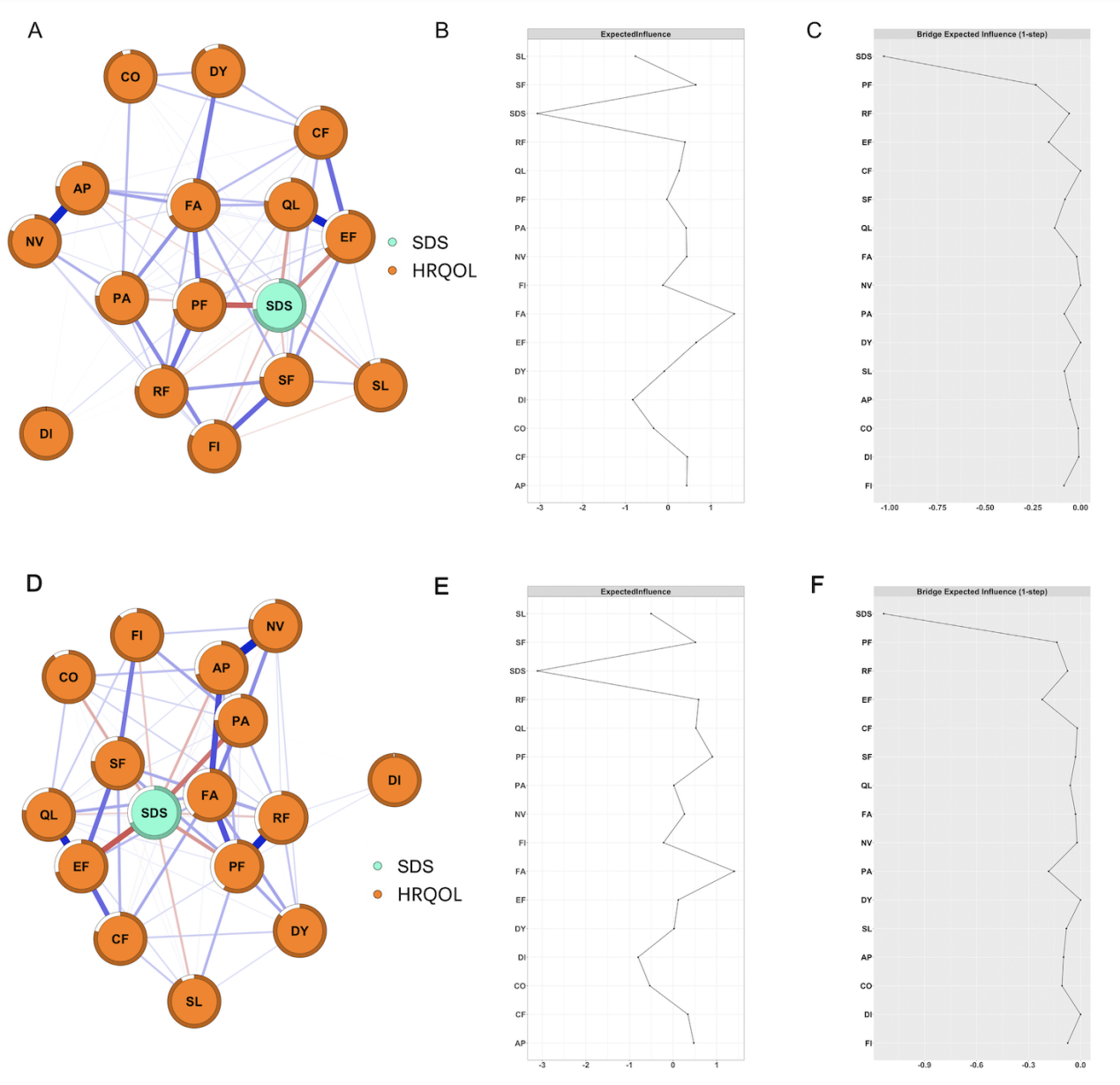


**Figure S9.**

**Comparison of network structure and centrality between whether surgery patients; (M = 0.111, p = 0.431); (S = 0.281, p = 0.215)**

**A Network structure; B Expected influnce; C Bridge Expected influnce: Received surgery;**

**D Network structure; E Expected influnce; F Bridge Expected influnce: Not received surgery**

**
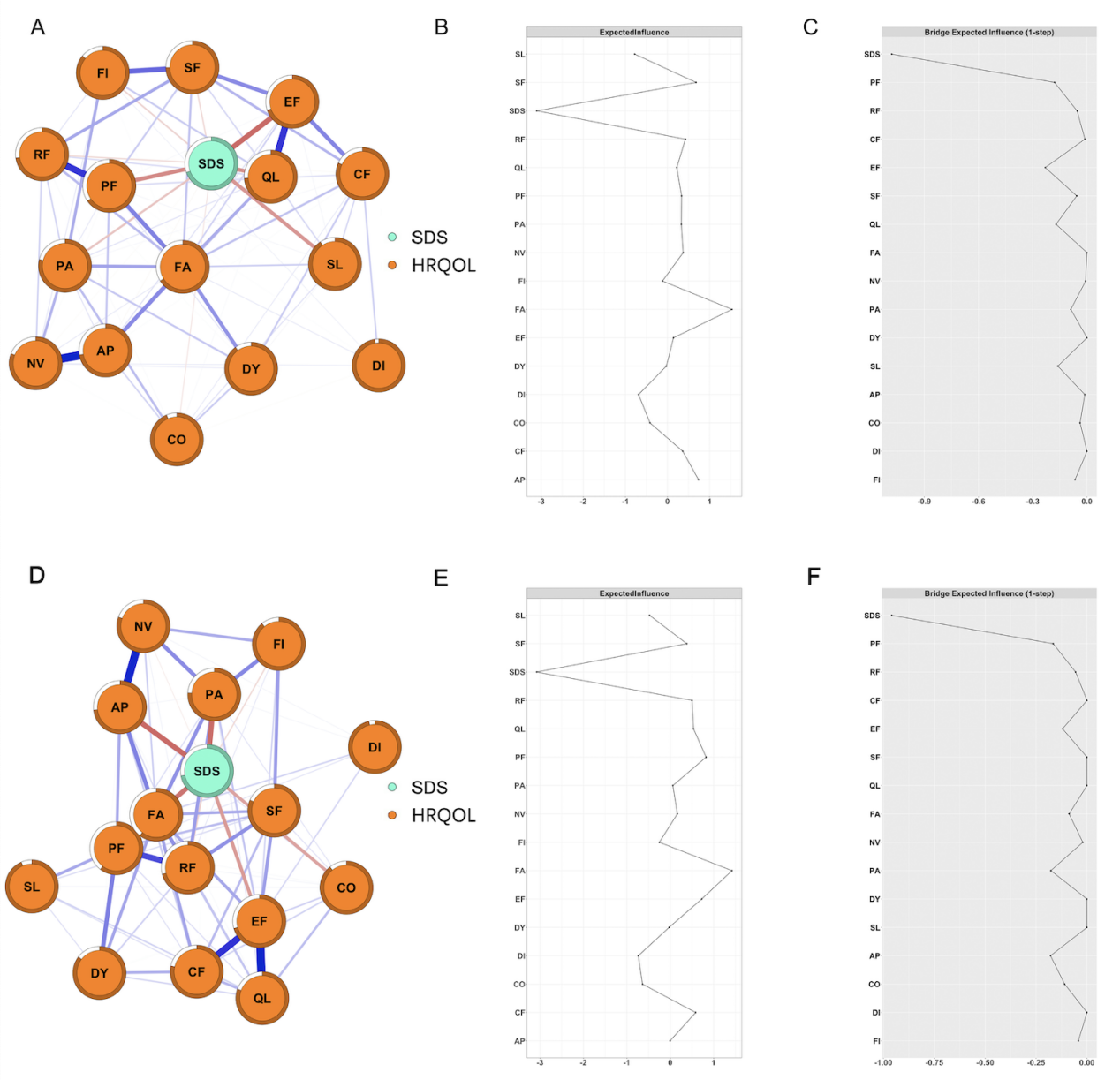
**

**Figure S10.**

**Comparison of network structure and centrality between whether chemotherapy patients; (M = 0.303, p = 0.532)(S = 4.478, p = 0.547)**

**A Network structure; B Expected influnce; C Bridge Expected influnce: Not received chemotherapy;**

**D Network structure; E Expected influnce; F Bridge Expected influnce: received chemotherapy**

**
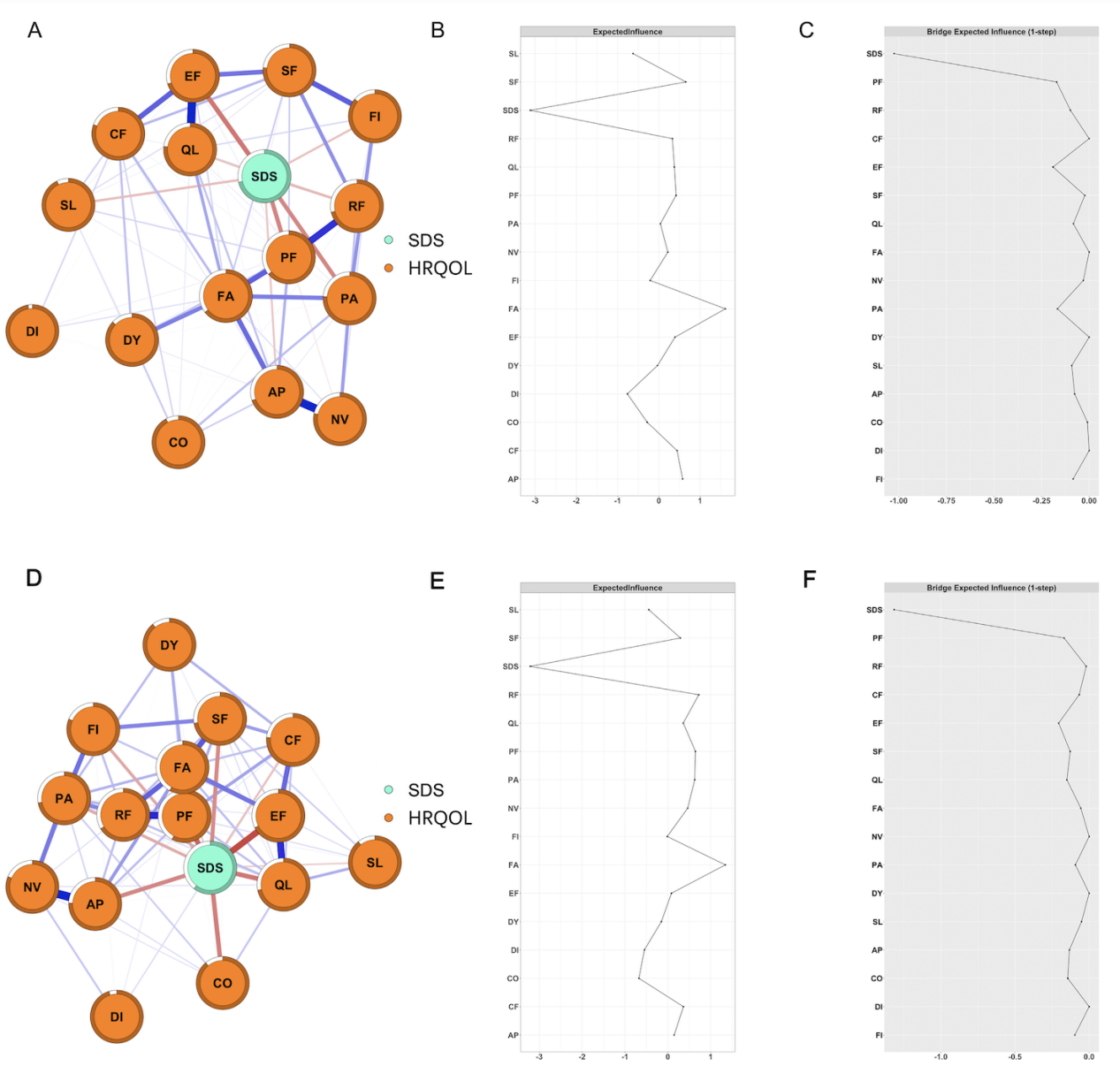
**

**Figure S11.**

**Comparison of network structure and centrality between whether radiotherapy patients; (M = 0.296, p = 0.599)(S = 3.439, p = 0.713)**

**A Network structure; B Expected influnce; C Bridge Expected influnce: Received radiotherapy;**

**D Network structure; E Expected influnce; F Bridge Expected influnce: Not received radiotherapy**

**
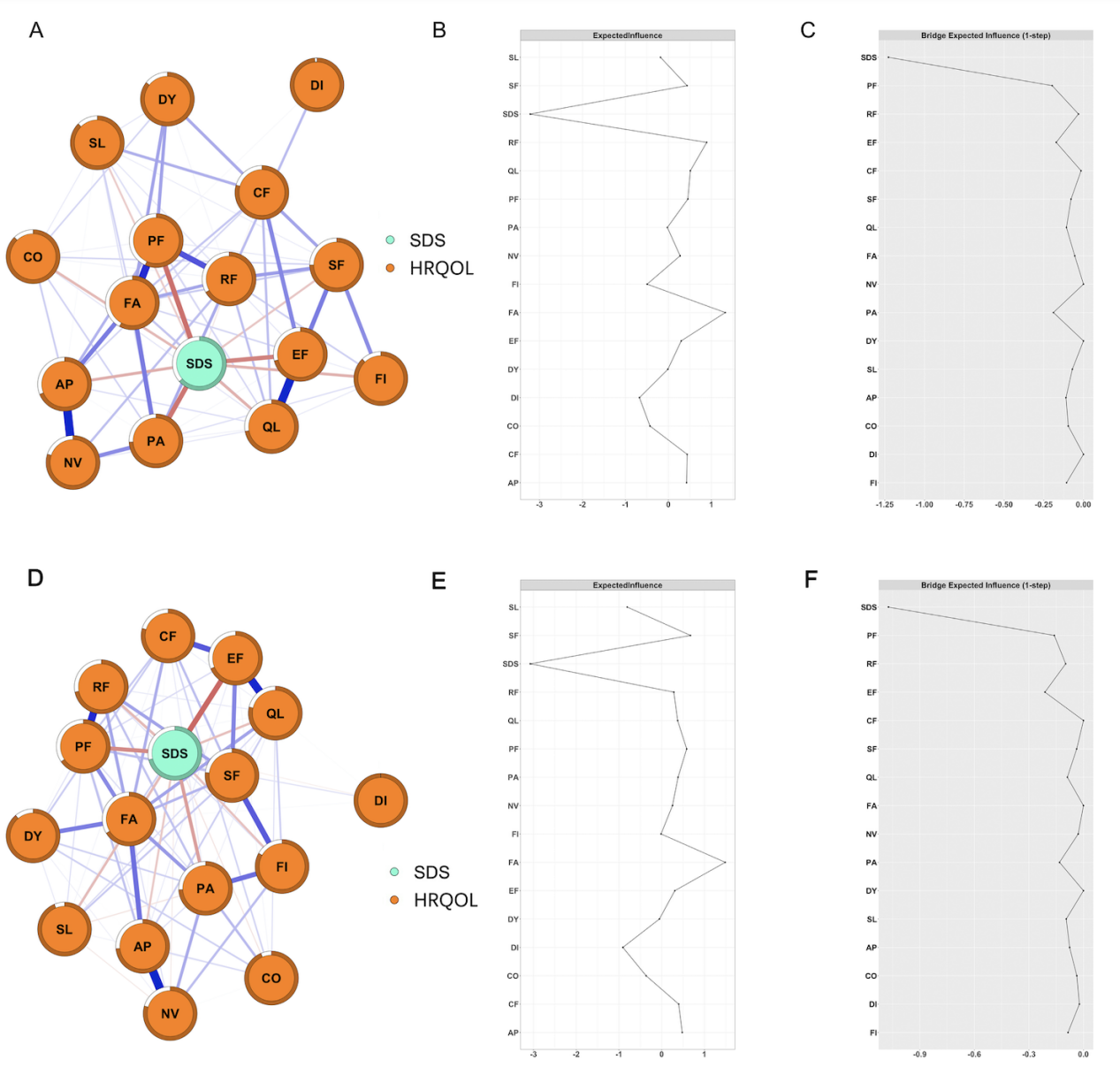
**

**Figure S12.**

**Comparison of network structure and centrality between patients which month from diagnosis<12Month and month from diagnosis≥12Month; (M = 0.128, p = 0.31)(S = 0.088, p = 0.787)**

**A Network structure; B Expected influnce,C Bridge Expected influnce : Month from diagnosis<12Month;**

**D Network structure; E Expected influnce,F Bridge Expected influnce: Month from diagnosis≥12Month;**

**
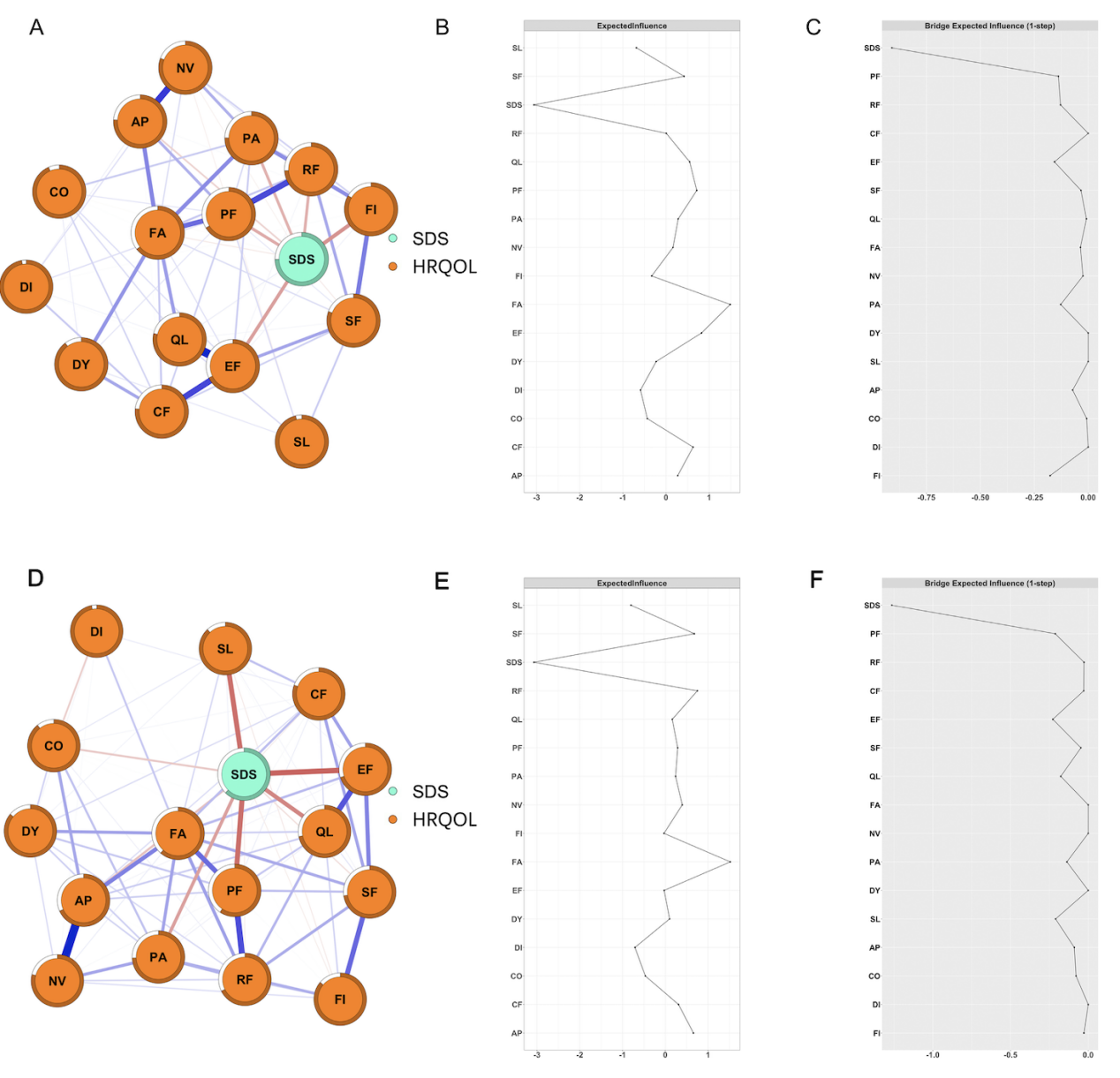
**

**Figure S13.**

**Network structure and expected influence for HRQOL dimensions of no depression patients:**

**A Network Structure;**

**B Expected Influence**

**
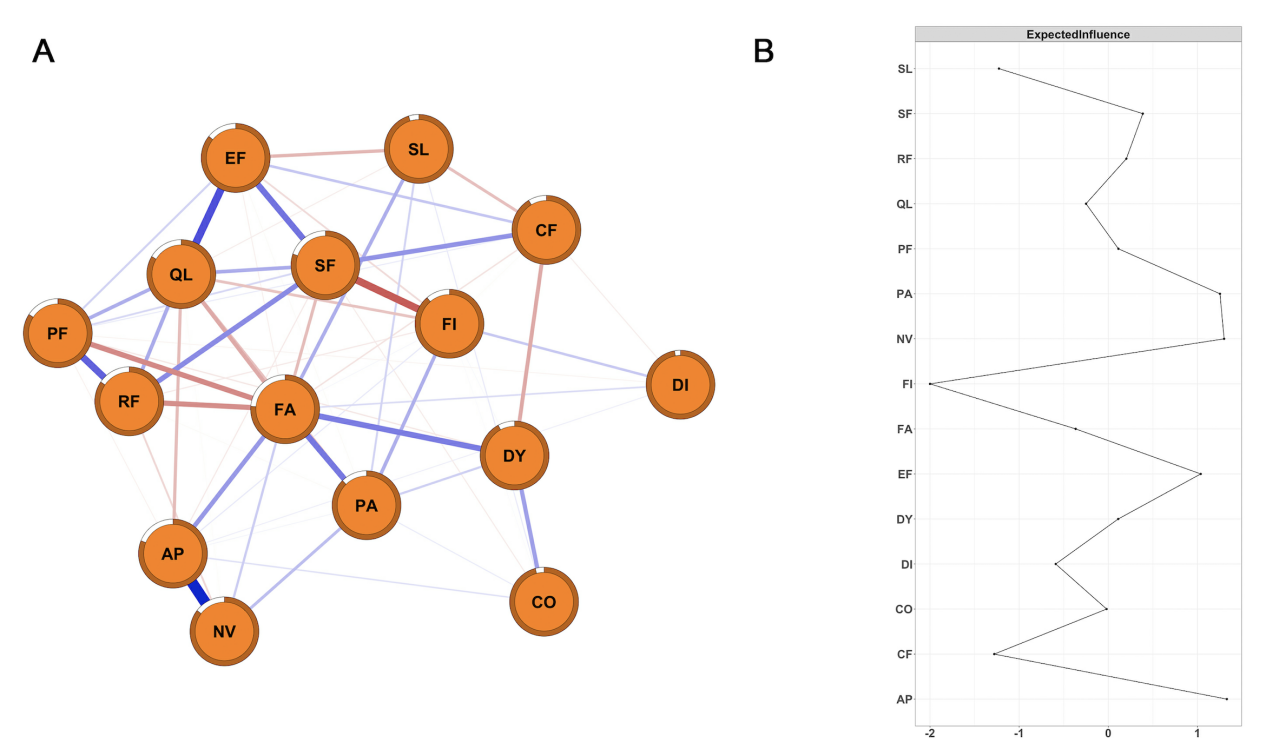
**

**Figure S14.**

**Directed acyclic graph (DAG) for HRQOL dimensions of no depression patients: (a) edge thickness represents the importance of that edge to the overall DAG structure; (b) edge thickness represents the directional probability**

**
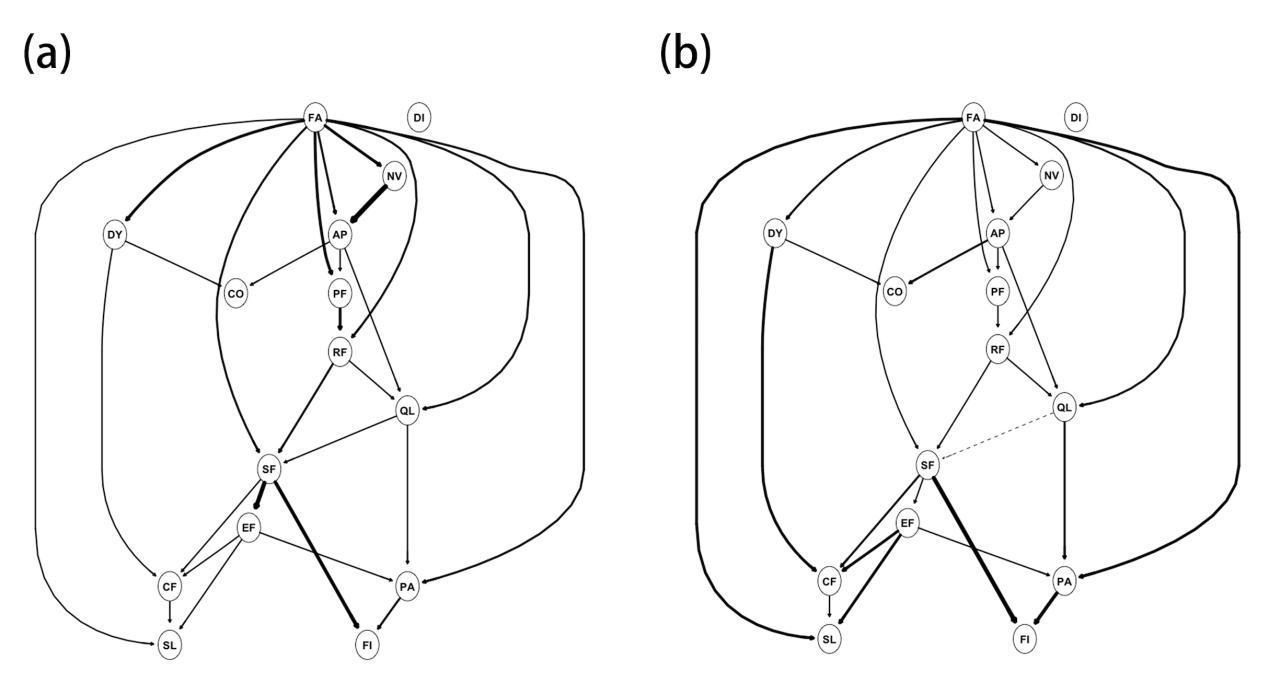
**

**Figure S15.**

**Estimation network structure of the relationship between depression and HRQOL in breast cancer**

**
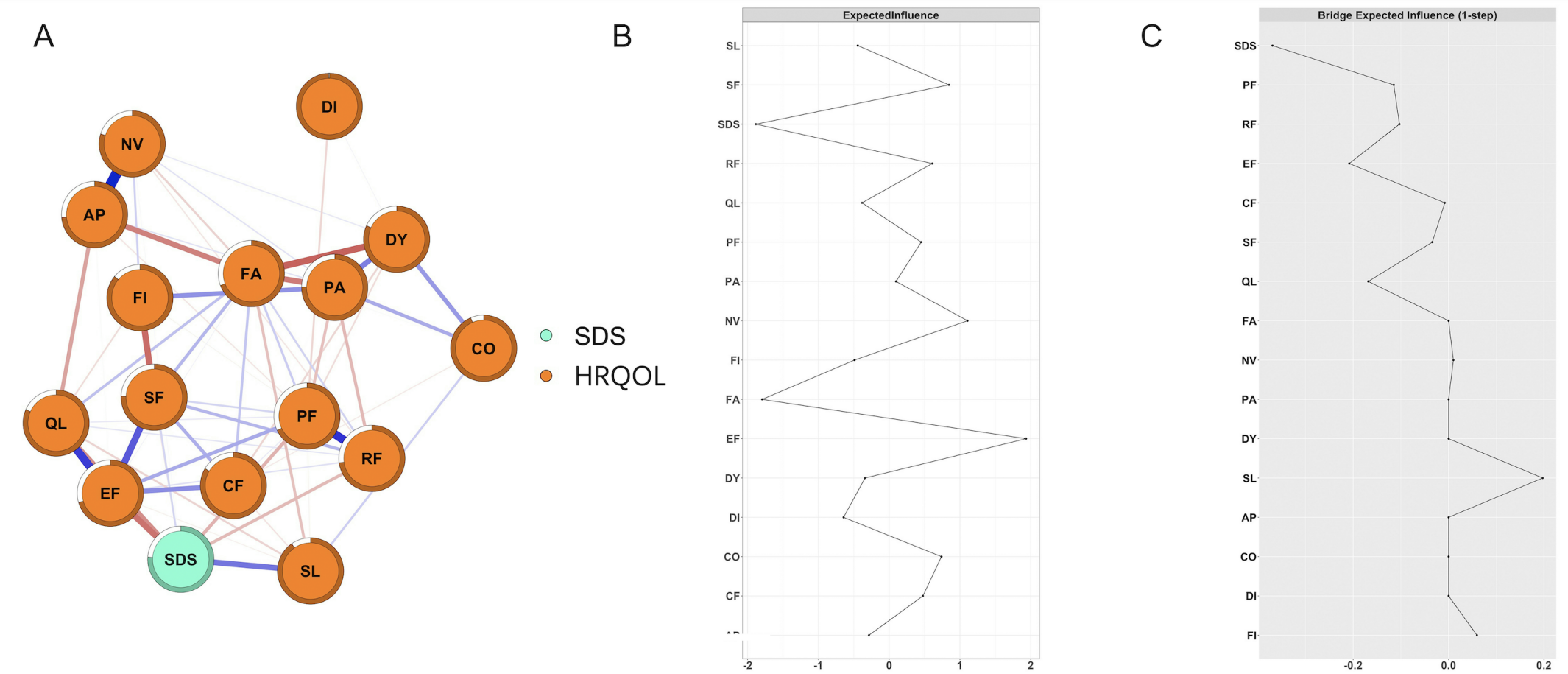
**

**Figure S16.**

**Estimation network structure of the relationship between depression and HRQOL in Colorectal cancer**

**
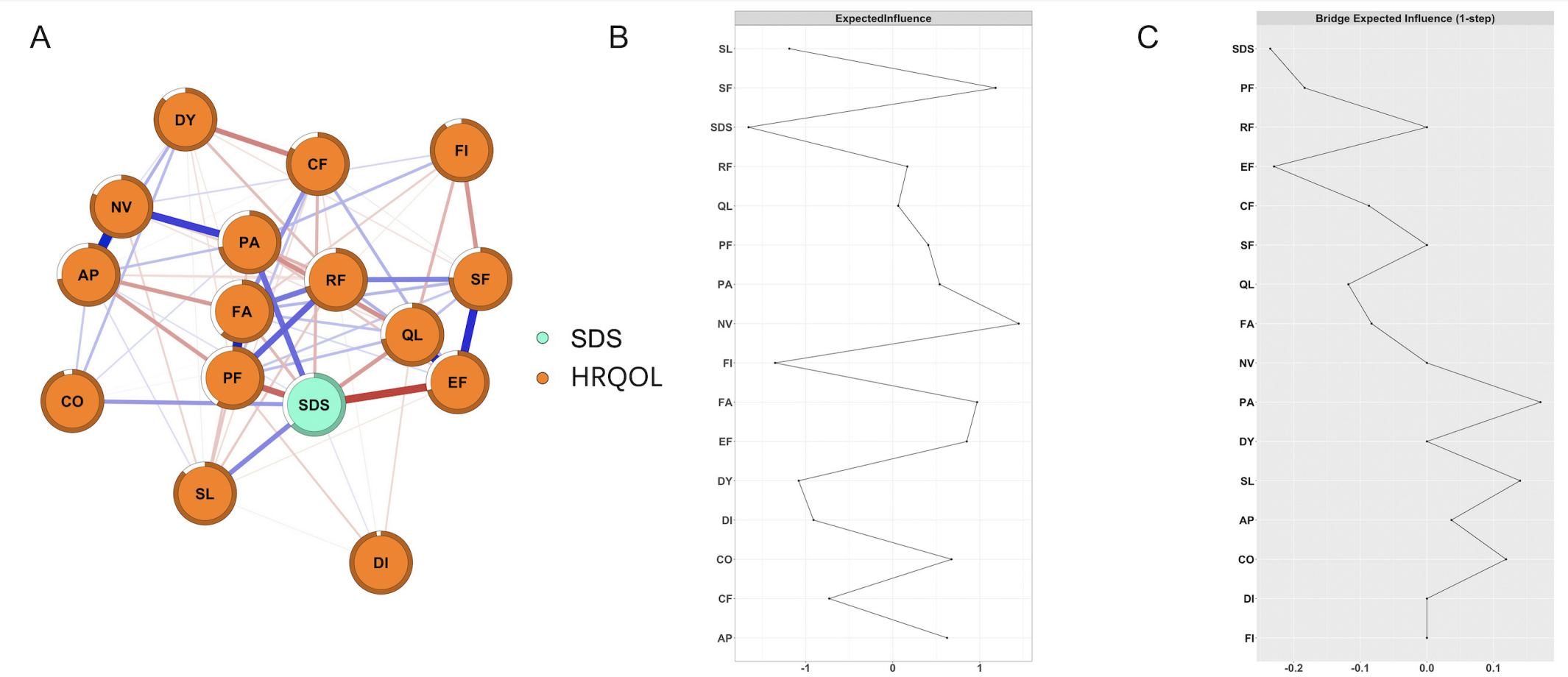
**

**Figure S17.**

**Estimation network structure of the relationship between depression and HRQOL in cervical cancer**

**
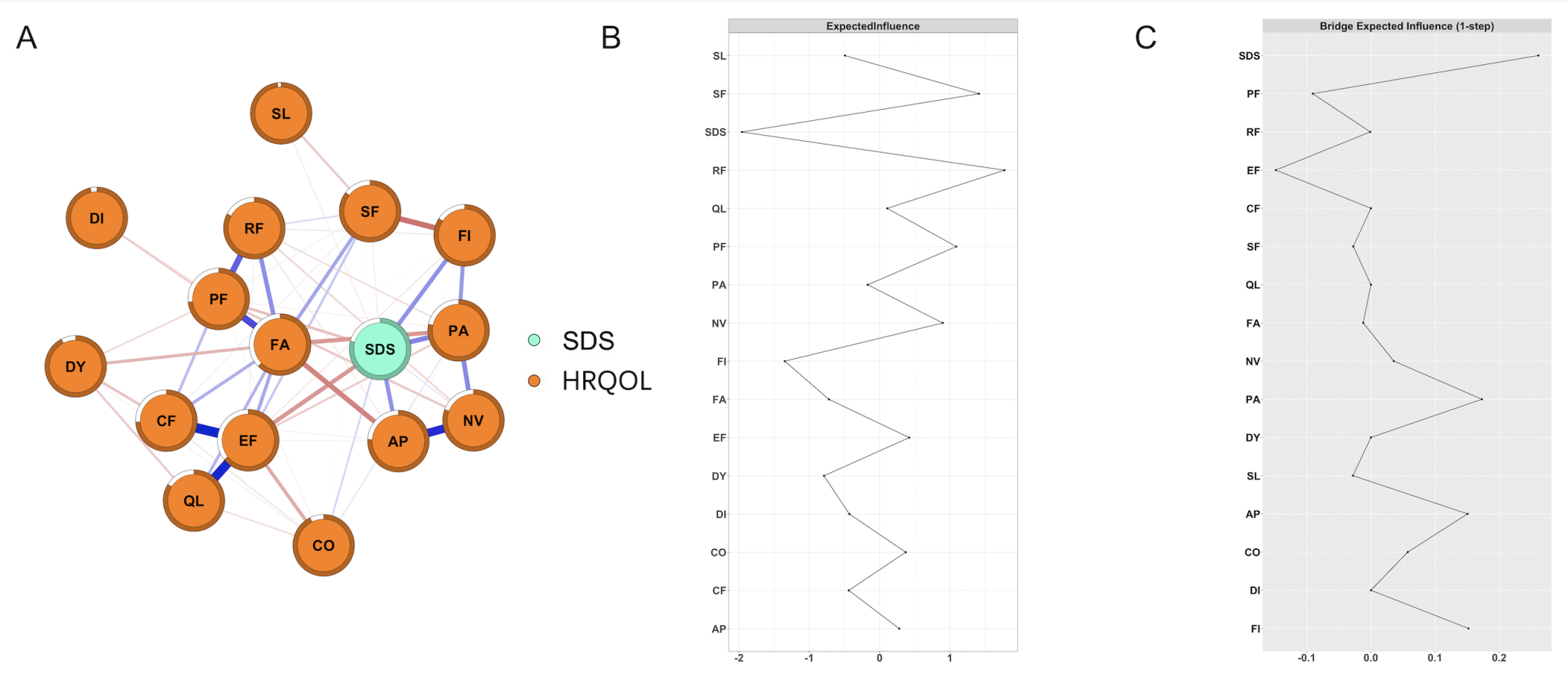
**

**Figure S18.**

**Estimation network structure of the relationship between depression and HRQOL in gastric cancer**

**
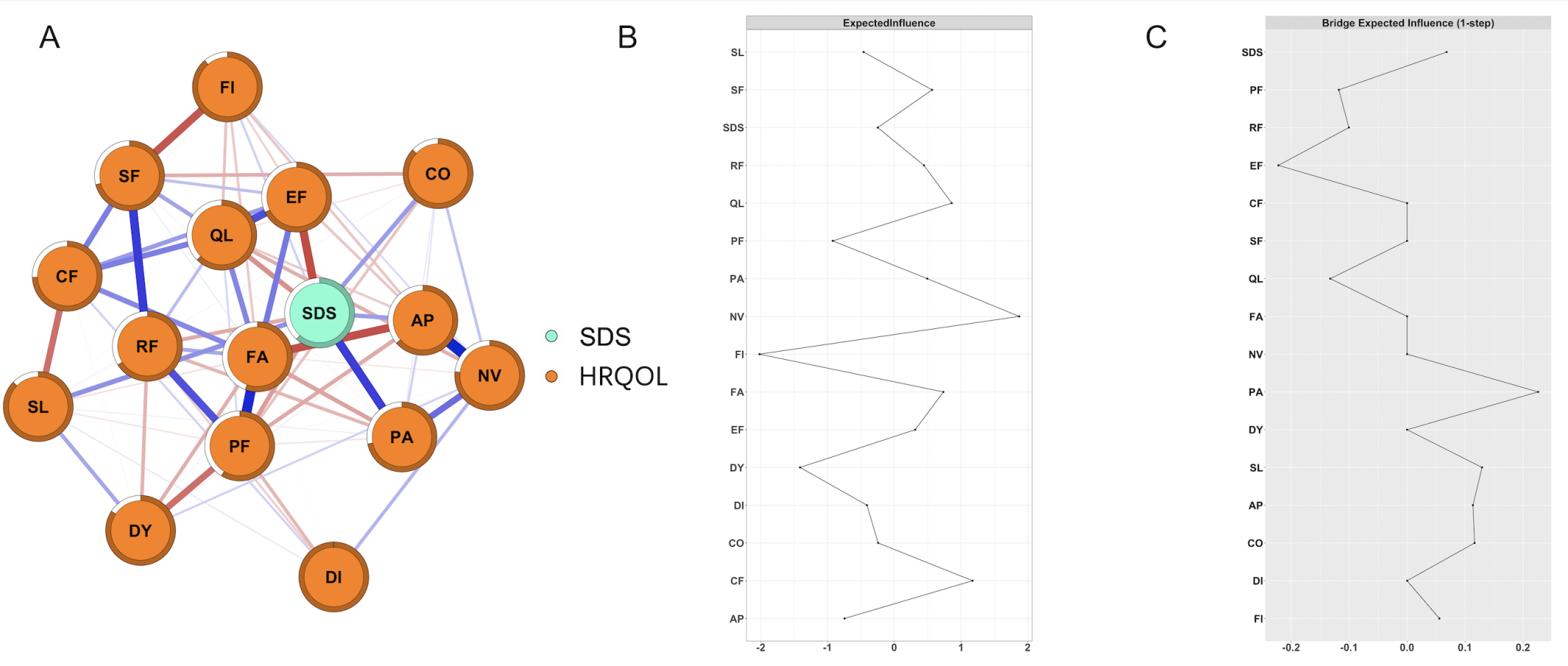
**

**Figure S19.**

**Estimation network structure of the relationship between depression and HRQOL in head and neck cancer**

**
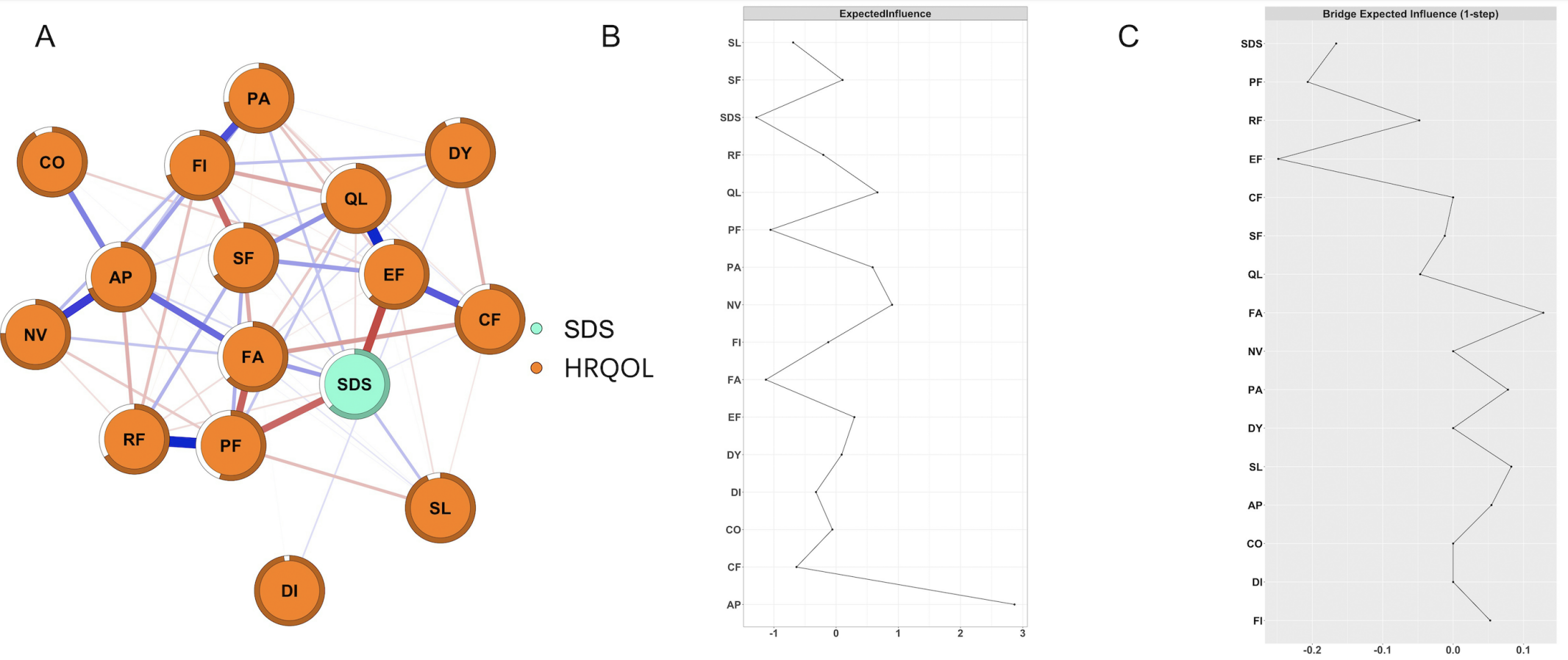
**

**Figure S20.**

**Estimation network structure of the relationship between depression and HRQOL in esophagus cancer**

**
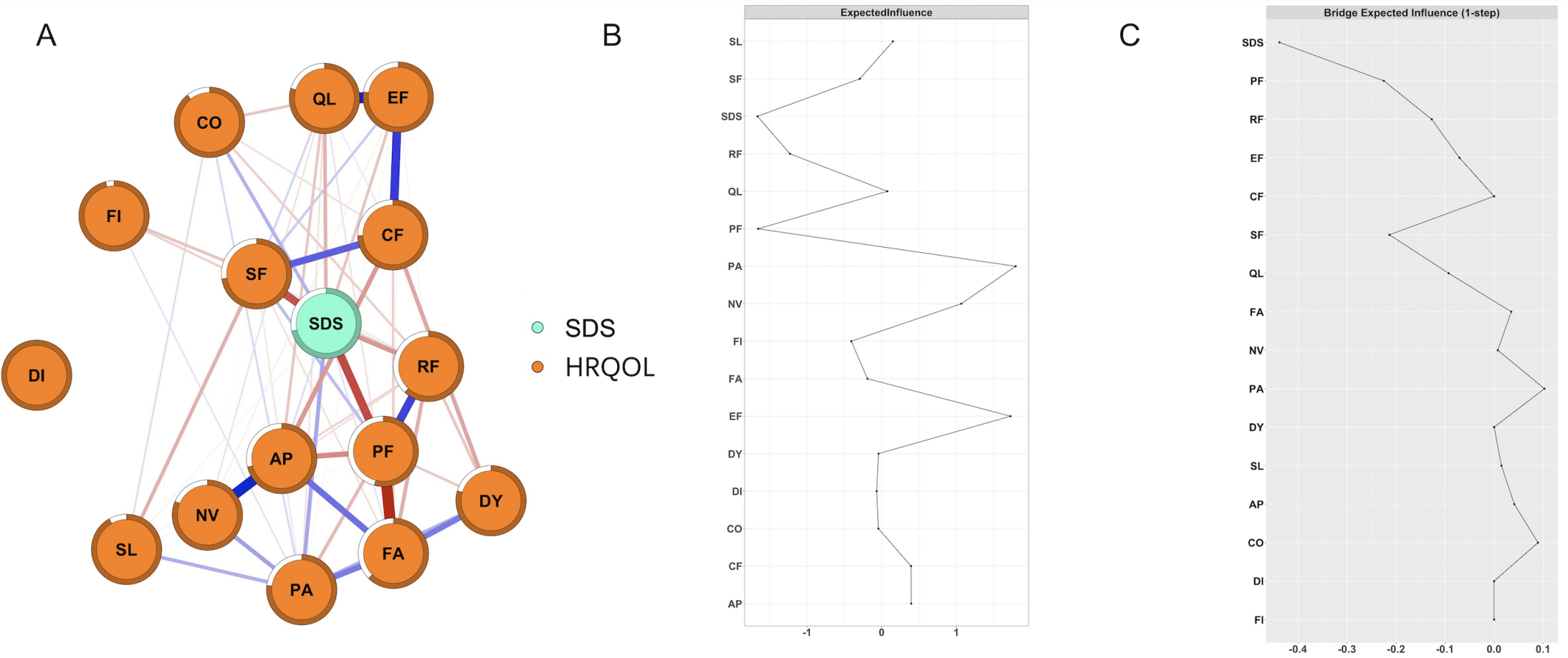
**
